# Selection maintains a nonadaptive floral polyphenism

**DOI:** 10.1093/evlett/qrae017

**Published:** 2024-04-25

**Authors:** José María Gómez, Adela González-Megías, Cristina Armas, Eduardo Narbona, Luis Navarro, Francisco Perfectti

**Affiliations:** Estación Experimental de Zonas Áridas (EEZA-CSIC), Almería, Spain; Research Unit Modeling Nature, Universidad de Granada, Granada, Spain; Research Unit Modeling Nature, Universidad de Granada, Granada, Spain; Departamento de Zoología, Universidad de Granada, Granada, Spain; Estación Experimental de Zonas Áridas (EEZA-CSIC), Almería, Spain; Departamento de Biología Molecular e Ingeniería Bioquímica, Universidad Pablo de Olavide, Sevilla, Spain; Departamento de Biología Vegetal y Ciencias del Suelo, Universidad de Vigo, Vigo, Spain; Research Unit Modeling Nature, Universidad de Granada, Granada, Spain; Departamento de Genética, Universidad de Granada, Granada, Spain

**Keywords:** adaptive plasticity, within-individual plasticity, natural selection, maladaptation, floral plasticity, pollinators

## Abstract

Adaptive phenotypic plasticity evolves in response to the contrasting selection pressures that arise when organisms face environmental heterogeneity. Despite its importance for understanding how organisms successfully cope with environmental change, adaptive plasticity is often assumed but rarely demonstrated. We study here the adaptive nature of the extreme seasonal within-individual floral polyphenism exhibited by the crucifer *Moricandia arvensis*, a Mediterranean species that produces two different types of flowers depending on the season of the year. During spring, this species has large, cross-shaped, lilac flowers, while during summer, it develops small, rounded, white flowers. Although floral polyphenism was associated with increased plant fitness, selection moved floral traits away from their local optimum values during the harsh summer. This result strongly suggests that floral polyphenism is not adaptive in *M. arvensis*. The main factor selecting against floral polyphenism was pollinators, as they select for the same floral morph in all environments. Despite not being adaptive, floral polyphenism occurs throughout the entire distribution range of *M. arvensis* and has probably been present since the origin of the species. To solve this paradox, we explored the factors causing floral polyphenism, finding that floral polyphenism was triggered by summer flowering. Summer flowering was beneficial because it led to extra seed production and was favored by adaptive plasticity in leaf functional traits. Taken together, our study reveals a complex scenario in which nonadaptive floral polyphenism has been indirectly maintained over *M. arvensis* evolutionary history by selection operating to favor summer flowering. Our study provides thus strong evidence that nonadaptive plasticity may evolve as a byproduct of colonizing stressful environments.

## Teaser

How organisms adapt to changing environments is a central question in ecology and evolution. Adaptive plasticity is a pervasive strategy used by organisms to successfully cope with environmental change. Here we study the adaptive nature of a within-individual floral polyphenism widely displayed by a Mediterranean weed. During spring, this species produces large, cross-shaped, lilac flowers, while during summer, it develops small, rounded, white flowers. Despite being associated with increased fitness, this floral plasticity was not adaptive because pollinators consistently prefer the same floral phenotype across environments and select against floral plasticity. The prevalence of this nonadaptive floral plasticity is an indirect consequence of plants benefiting from flowering under stressful conditions that prevent the expression of optimal floral phenotypes. We provide strong evidence that nonadaptive plasticity may evolve when organisms colonize and establish in stressful conditions.

## Introduction

How organisms adapt to changing environments is a central question in ecology and evolution (Levins, 1968; Pfennig, 2021). Phenotypic plasticity, the ability of a genotype to produce alternative phenotypes when exposed to different environments, is a pervasive response of most organisms when facing varying conditions (Nylin & Gotthard, 1998; Schlichting & Pigliucci, 1998). Although the plastic response of certain phenotypic traits may only be the consequence of environmentally induced passive effects on those traits (Brooker et al., 2022; Schneider, 2022; Van Kleunen & Fischer, 2005), organisms often respond actively to environmental variations to avoid any reduction in fitness (Brooker et al., 2022; Ghalambor et al., 2007). In fact, phenotypic plasticity might elicit the emergence of novel phenotypes with new adaptive possibilities, which may confer selective advantages in some contexts (Pfennig, 2021; Snell-Rood & Ehlman, 2021; Sultan, 2021). Since phenotypic plasticity is ubiquitous and, in many cases, enables organisms to cope successfully with environmental changes, it is widely assumed that plastic responses are adaptive (Bonser, 2021; Sultan, 2000). However, demonstrating the adaptive value of plasticity, while fundamental to understanding how plasticity evolves, is a conceptual and methodological challenge (Alper & Simms, 2002; Pfennig, 2021; Sultan, 2021; van Buskirk & Steiner, 2009; Van Kleunen & Fischer, 2005; Via, 1993). Consequently, the extent and strength of adaptive plasticity in natural systems remains still an open question (Arnold, Nicotra, et al., 2019; Ghalambor et al., 2007; Palacio-López et al., 2015).

Three non-exclusive views have been developed to assess the adaptive nature of plasticity. First, plasticity is considered adaptive if it involves a net fitness gain (Alper & Simms, 2002; Arnold, Nicotra, et al., 2019; Bonser, 2021; Gotthard & Nylin, 1995; Pfennig, 2021; Pigliucci, 2001). However, providing fitness benefits, although necessary, is not enough to confer adaptive value to plasticity. This is so because fitness advantages can be caused not only by the direct effect of natural selection on plastic traits but also by the correlation with other traits that are the true targets of selection (Via & Lande, 1985). Determining the adaptive nature of plasticity thus requires understanding the patterns of selection acting on the plastic traits (Gomulkiewicz & Kirkpatrick, 1992; Pigliucci & Schlichting, 1996; Stearns, 1989; Via & Lande, 1985; Weis & Gorman, 1990).

A second view considers plasticity as a trait that is itself the object of selection and evolves independently of trait values (DeWitt, 1998; Gomulkiewicz & Kirkpatrick, 1992; van Buskirk & Steiner, 2009; Via, 1993). Under this conception, plasticity is adaptive when selection acts to increase the absolute value of the slopes of reaction norms (Arnold, Kiruuk, et al., 2019; Arnold, Nicotra, et al., 2019; Gotthard & Nylin, 1995; van Buskirk & Steiner, 2009). This view has been prolific, and many studies have considered plasticity to be subject to selection (Arnold, Nicotra, et al., 2019; Blanco-Sánchez et al., 2023; Gotthard & Nylin, 1995; Stinchcombe et al., 2004; van Buskirk & Steiner, 2009; Van Kleunen & Fischer, 2005; Weis & Gorman, 1990). However, it can sometimes provide an incomplete or ambiguous description of the evolution of plasticity. Proposing that selection operates on an across-environment averaged plastic trait contradicts the widely assumed idea of selection as a process that operates locally to optimize trait values within each environment (De Jong, 2005; Gomulkiewicz & Kirkpatrick, 1992; Via, 1993; Via & Lande, 1985). Selection operating directly on dimensionless slopes of the reaction norms cannot distinguish among genotypes with parallel reaction norms but different trait values and fitness effects (De Jong, 2005; Schneider, 2022) or among those phenotypes directly driven by growth-limiting resource shortage in response to stressful environments but without any functional consequence (Brooker et al., 2022).

A third approach considers adaptive plasticity to evolve due to contrasting selection operating on traits in each environment rather than on plasticity itself (Bradshaw, 1965; De Jong, 2005; Ghalambor et al., 2007; Gomulkiewicz & Kirkpatrick, 1992; Murren et al., 2015; Scheiner, 2013; Via, 1993; Via & Lande, 1985). The targets of selection are, under this approach, the within-environment traits. Because natural selection drives population mean phenotypes toward their local optimal values (Endler, 1986; Lynch & Walsh, 1998), plasticity can be considered adaptive when it moves the trait closer to these optimal values in each environment (Nussey et al., 2007; Palacio-López et al., 2015; Van Tienderen, 1991). When adaptive plasticity produces a near perfect match with the optimal phenotype in the new environment, the population should also experience stabilizing selection with no subsequent genetic differentiation between populations (Palacio-López et al., 2015). Under this perspective, plasticity is adaptive when within-environment selections occur in opposite directions and are concordant with across-environment plastic differences in trait values (Caruso et al., 2006).

In this study, we evaluate the adaptive value of the floral polyphenism expressed by the mustard species *Moricandia arvensis* (Brassicaceae) by means of the three complementary methods described above. Flowers are highly integrated structures made up of multiple coevolved parts that function together in a coordinated manner to attract effective pollinators and promote plant reproduction (Glover, 2014; Harder & Barrett, 2006). The environmentally induced modification of single floral traits may imperil the correct functioning of the entire structure and diminish the fitness of the overall phenotype. Because the sensitivity of a trait to environmental perturbations is proportional to its impact on fitness (Klingenber, 2019; Wagner, 1997), flowers tend to show high developmental canalization (Pélabon et al., 2011), expressing plasticity less frequently than other plant traits and affecting only some quantitative floral parts (Rusman et al., 2019; Sultan, 2000). *Moricandia arvensis* is exceptional because plasticity is expressed at the level of the entire flower. Thus, this species bears radically different flowers during the temperate and humid spring and the extremely dry and hot summer of the Western Mediterranean drylands, its native distribution range (Gómez et al., 2020, 2022). During spring, this species has large, cross-shaped, lilac flowers, similar to the canonical flowers of most other *Moricandia* species, while during summer, it has small, rounded, white flowers, similar to those produced by other species belonging to distant lineages (Supplementary Figure S1). More remarkably, this multivariate plasticity is expressed intraindividually, with the same plant changing its floral phenotype from spring to summer (Gómez et al., 2020). This means that selection on floral plasticity acts through the same genotypes across environments. We test here whether the floral polyphenism exhibited by *M. arvensis* is adaptive, with individuals expressing those floral phenotypes that maximize seed production in each environment.

## Methods

### The adaptive value of plasticity

We explored the adaptive value of plastic traits by determining in 100 co-occurring plants from one natural population (Negratín population, Supplementary Table S1, Supplementary Dataset S1), the fitness difference of plastic and nonplastic individuals, the direction and strength of the selection differential occurring each season on 12 floral traits describing the floral polyphenism (Supplementary Table S2), and the selection occurring on the magnitudes of their plasticity (see below for definitions and calculations of each selection parameter). In order to check whether selection may affect floral plasticity indirectly through other nonfloral traits, we repeated these analyses for six leaf economics spectrum (LES) traits and four life-history (LH) traits (Supplementary Table S2) (see Supplementary Methods S1 for assessment of plant phenotypic traits).

#### Quantification of within-individual plasticity

We used random slope mixed models to estimate the significance and magnitude of the within-individual plasticity of each *M. arvensis* trait (Dryden & Mardia, 2016). We built a first model including as fixed effect the mean-centered average daily temperature calculated as the seasonal temperature weighted by the photoperiod (Supplementary Table S1). This model was used to assess the population-level effect of temperature on the value of each trait (population-level plasticity). We ran a second model including the identity of each individual plant as a random effect. We used this model to estimate the significance of among-individual variation in trait values. For this, we compared between the second and first models the goodness-of-fit by means of the Akaike information criterion (AIC) and the log-likelihood by means of a likelihood ratio test (Arnold, Kiruuk, et al., 2019). We ran a third model adding an extra random regression term to estimate the significance of the among-individual differences in the slopes of their reaction norms (the occurrence of G × E interaction). For this, we proceeded as above and compared the AIC and log-likelihood of the third and second models (see Supplementary Methods S2 for analytical details).

#### Fitness benefit of plasticity

To evaluate the benefits in term of fitness of floral polyphenism, we compared the total and seasonal production of seeds per plant between plants flowering during summer and expressing floral polyphenism and those flowering only in spring and expressing one single floral form in the Negratín population. In addition, we marked 100 plants in each of five populations of SE Spain (Supplementary Table S1) to check the proportion of plants expressing floral polyphenism.

#### Selection on trait values within each environment

The total directional selection occurring on each phenotypic trait during each season was estimated by calculating the magnitude and sign of the selection differential (Arnold & Wade, 1984; Lande & Arnold, 1983; Lynch & Walsh, 1998). Fitness was calculated as the seasonal production of seeds per plant. That is, in this model, fitness was environment specific. The statistical significance of the selection differentials was calculated by fitting univariate linear models (Lande & Arnold, 1983; Lynch & Walsh, 1998). We also checked for the occurrence of stabilizing selection on plant traits within each environment by fitting univariate quadratic models (Lande & Arnold, 1983; Lynch & Walsh, 1998) (see Supplementary Methods S2 for analytical details).

#### Selection on the slopes of the reaction norms

The selection occurring on the across-environment plasticity of each phenotypic trait was estimated using mixed models (Arnold, Kiruuk, et al., 2019). We performed a simple linear model relating the total relative fitness of each genotype *i* combining all environments with the slope of each plastic trait. Total relative fitness was calculated as the sum of seeds produced by each genotype in each environment. The slope of the reaction norm of each genotype was obtained by performing random regression mixed models as explained above and using the BLUP slopes as an estimate of the deviance of the plasticity of each genotype from the population-level plasticity (Arnold, Kiruuk, et al., 2019). However, it is widely known that the slope of the reaction norm is correlated with the average values of the trait for most plastic traits (Arnold, Nicotra, et al., 2019). For this reason, we performed a second multivariate mixed model in which we controlled for this covariance by including in the model not only the slope but also the average value of the trait (Stinchcombe et al., 2004; Van Kleunen & Fischer, 2005). The average value of each trait was calculated as the individual BLUP intercepts of the random regression mixed models, as explained above (Arnold, Kiruuk, et al., 2019) (see Supplementary Methods S2 for analytical details).

### Determination of the selective agents affecting floral polyphenism

We explored the role of pollinators as selective agents mediating selection on floral plasticity by mean of structural equation modeling (SEM). Detailed methods about how we assessed the traits and the abundance of pollinators at flowers are described in Supplementary Methods S1 and S3. A total of 26 pollinator functional groups visited the flowers of *M. arvensis* during the study period, although only seven groups accounted for more than 1% of the floral visits in any of the two seasons (long-tongued large bees, short-tongued large bees, short-tongued medium-sized bee, short-tongued small bees, large butterflies, large beeflies, small diving beetles; Supplementary Table S3). We included in the SEMs the abundance at flowers of these pollinator functional groups as well as the slope (in absolute values) of the reaction norms of the 12 floral traits (Supplementary Dataset S1). We used as fitness estimate the lifetime seed production of each individual by combining the seeds produced during spring and summer. Using the information provided by the selection analyses, we built an a priori overidentified saturated model in which plant fitness was directly connected to the main seven floral visitor functional groups and to the plasticity of floral traits. We solved the SEMs by building a set of alternative nested models where we constrained some of the causal paths to zero. In these models, the total path coefficients generated by the SEMs can be interpreted as the total selection acting on the plasticity exhibited by each phenotypic trait (Scheiner et al., 2000). All models were solved by minimizing yield-parameter estimates through an iterative process that uses generalized least squares shifting to maximum likelihood as discrepancy functions. We used maximum-likelihood estimation on the variance–covariance matrix to test the goodness of fit of the models. We retained those models obtaining an appropriate goodness of fit (*p* > 0.05, Grace, 2006). We then checked their standardized root mean square residual (SRMR), their root mean square error of approximation (RMSEA), and their comparative fit index (CIF). SRMR and RMSEA < 0.05 indicates a good fit to data and between 0.05 and 0.1 indicates an acceptable fit. CFI > 0.97 means that the fit is better compared to the independence model (Cangur & Ercan, 2015). SEM was performed using the R package lavaan (Rosseel, 2012).

### Estimating the cost of floral plasticity

We checked whether the evolution of floral plasticity could be constrained in *M. arvensis* due to the existence of plasticity costs. Using the approach proposed by Scheiner and Berrigan (1998), we estimated maintenance and production costs of plasticity as the negative value of the regression coefficient of genotype relative environment-specific fitness on genotype plasticity (Dorn et al., 2000; Scheiner & Berrigan, 1998; van Buskirk & Steiner, 2009). When the cost was significant, we estimated additional production costs by including in the previous model the interaction between the expression of the trait in each environment and the slope of the trait plasticity (Scheiner & Berrigan, 1998). This additional cost of plasticity is detected when the interaction term has a significantly negative value (Scheiner & Berrigan, 1998) (see Supplementary Methods S2 for analytical details).

### Factors mediating the expression of floral polyphenism

We checked the effect of genetic factors by assessing the genetic similarity (based on seven microsatellites loci; Supplementary Dataset S2) between plants flowering during summer against those not flowering. Detailed genetic methods are described in Supplementary Methods S4. To explore ecological factors mediating floral polyphenism, we measured in each plant spring LES traits, spring LH traits, and herbivory-mediated spring stresses (Supplementary Tables S2 and S4). Detailed methods about how we assessed the traits and the impact of herbivores are described in Supplementary Methods S1 and S3. We related these three groups of variables (LES traits, LH traits, Herbivory) and the probability of flowering during summer by means of SEM with latent constructs (Grace, 2006). In these models, we connected LES to herbivory and LH, herbivory to LH, and LH to the probability of summer flowering. Detailed methods on how we built the models and how we solved them are described in Supplementary Methods S5.

## Results

### The adaptive value of plasticity

#### Quantification of within-individual plasticity

Plasticity was significant for all floral traits except two of the four geometric morphometric components of the corolla shape (*p* < 0.05 in all cases except for Corolla shape components 1 and 3; Supplementary Table S5). Likewise, plasticity was significant for all LES traits (*p* < 0.00001 in all cases; Supplementary Table S5). In contrast, most LH traits did not express significant plasticity (*p* > 0.1 in all cases except for number of ovules; Supplementary Table S5). The genotype × environment interactions were also significant for all traits (*p* < 0.004 in all cases; Supplementary Table S5), suggesting that natural selection can operate on the plastic component of all of them.

#### Fitness benefit of plasticity

**Figure 1.**
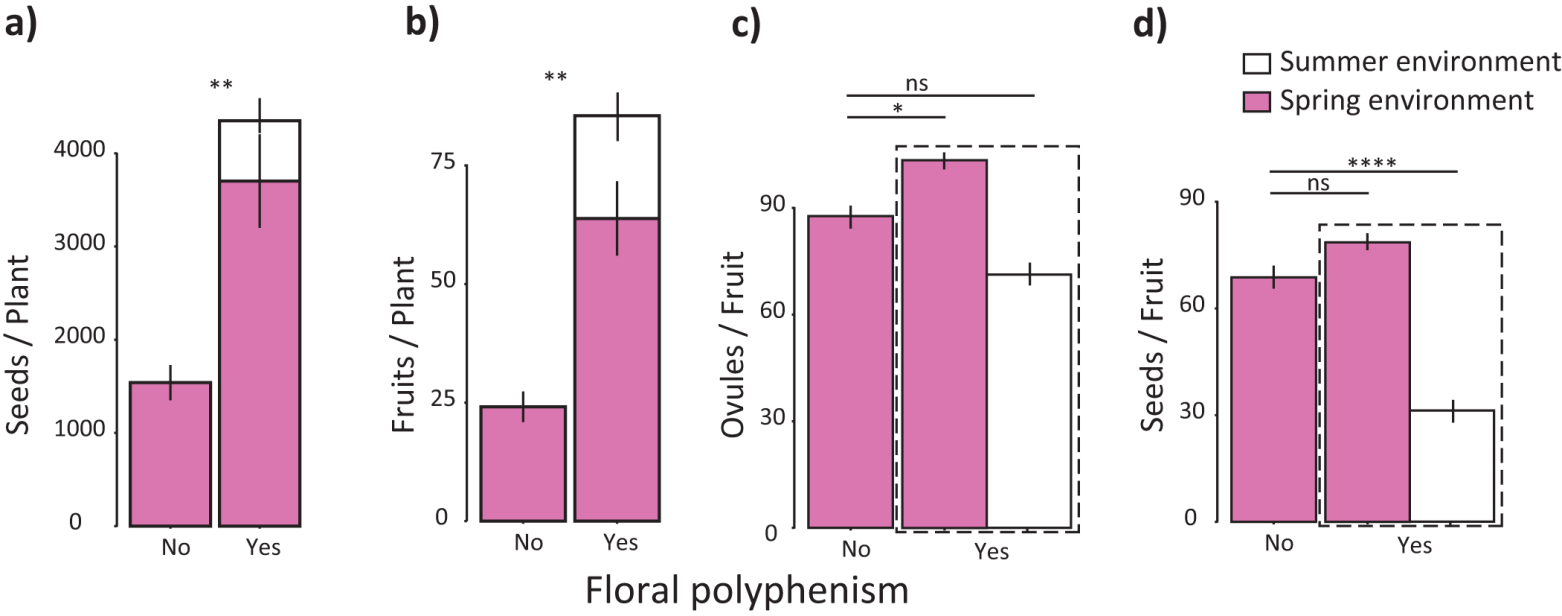
Fitness gain of plasticity. Fitness gain of plastic individuals quantified as (A) seed production. (B) Fruit production. (C) Ovule production per fruit and (D) Seeds produced per fruit. In (C) and (D), we compared both spring and summer fruits of summer-flowering plants against spring fruits of plants that did not flower during summer and produced flowers only during spring.

Although most plants can stay alive during summer, not all of them can produce flowers during this hot season. The proportion of plants expressing floral polyphenism ranged from 44% to 80% (500 plants, 5 populations; Supplementary Table S1). Floral polyphenism was invariably associated with flowering during summer. Although plants flowering in both seasons produced fewer flowers during summer (24 ± 5 flowers/plant, *N* = 100 plants) than during spring (387 ± 67 flowers/plant), floral polyphenism entailed a significant fitness gain in *M. arvensis* (Figure 1, Supplementary Table S6). Polyphenic plants produced 4,350 ± 596 seeds (mean ± 1 *SE*, *N* = 76 plants), whereas those plants bearing only one floral morph produced 1,539 ± 189 seeds (*N* = 24 plants; Figure 1A). This gain was mostly due to an extra production of fruits from summer-flowering plants both during summer and during spring (Figure 1B). In addition, seed production per fruit during summer, although lower than the number of seeds per fruit produced during spring, also contributed to the increased fitness of summer flowering plants (Figure 1D).

#### Selection on trait values within each environment and on the slopes of the reaction norms

**Figure 2.**
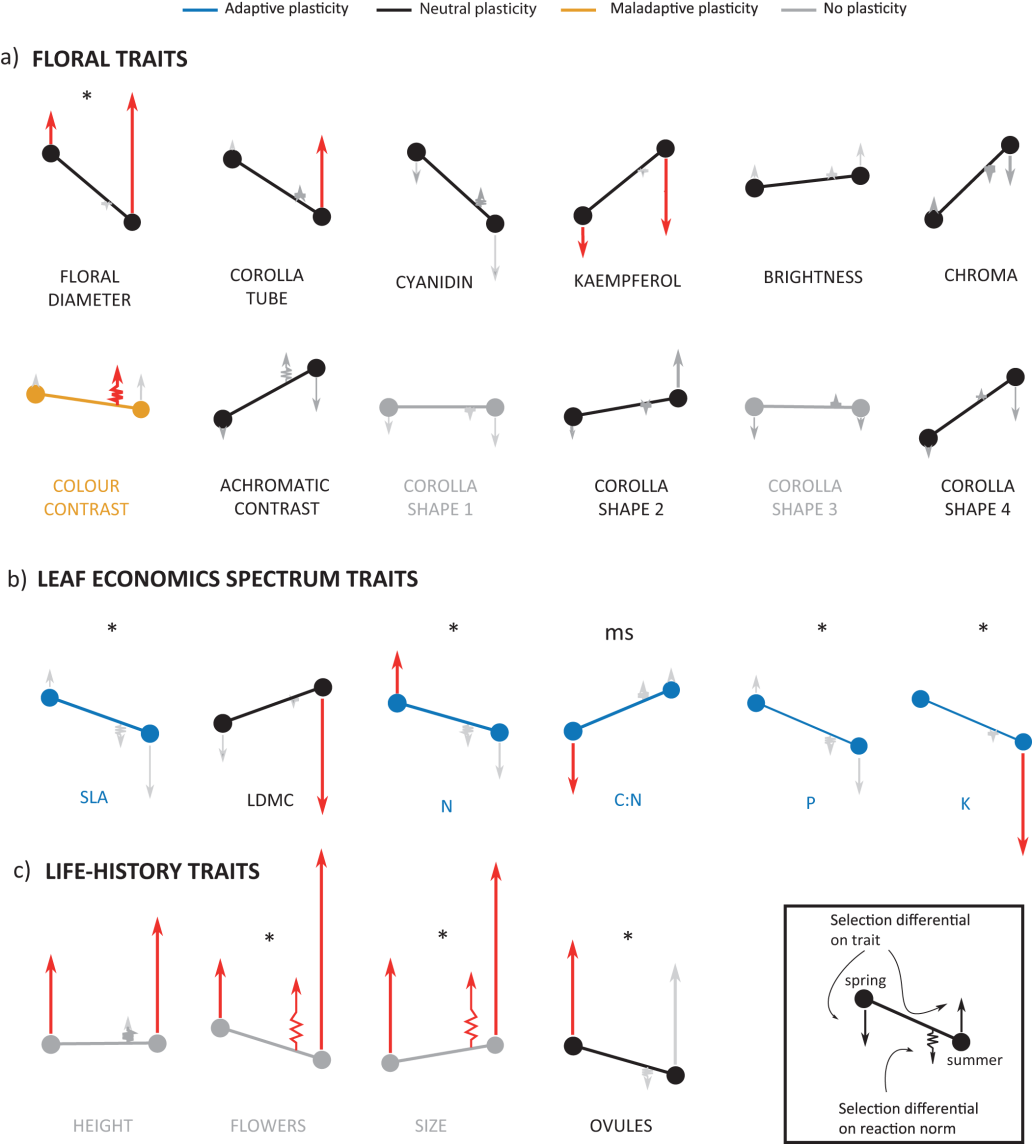
Adaptive value of floral plasticity in *Moricandia arvensis*. Magnitude and direction of the selection differentials acting on each plant trait during each season (vectors in arrow) and on their reaction norms (spring vectors). The reaction norms are represented as lines joining the phenotypic value in spring and summer. The spring vectors indicates selection acting directly on plasticity, increasing it (raising the slope) or decreasing it (flattening the slope). The length of the vectors indicates the magnitude of the selection, and the color, their statistical significance (gray = nonsignificant, red = significant). The asterisks above each reaction norm indicate significant between-season differences in the magnitude of selection. The color codes in reaction norms are: Blue indicates adaptive plasticity, orange indicates maladaptive plasticity, black indicates adaptatively neutral plasticity, and gray indicates nonsignificant plasticity.

The selection differential was significant only for three floral traits (floral diameter, floral corolla tube, and kaempferol content). Most importantly, the directions of the selection differentials were similar between seasons for most floral traits, indicating that the same floral trait values were selected both in spring and in summer (Figure 2A; Table 1). In particular, selection favored in summer the trait values expressed during spring (Figure 2A; Table 1). So, although floral diameters or corolla tubes were smaller in summer than in spring, selection favors larger values in both seasons (Figure 2A). Likewise, we found something similar for kaempferol content, which was higher during summer than during spring, but selection favors lower values in both seasons (Figure 2A). In addition, no quadratic selection differential was significant for any floral trait, suggesting no stabilizing selection on floral polyphenism in any of the environments (Supplementary Table S7). It seems that plasticity moved all floral traits away from their optima values, mostly because spring floral traits were favored by selection even in summer. The selection operating on the slopes of the floral reaction norms was consistent with these outcomes since, in most cases, selection acted flattening the reaction norms (Figure 2A; Table 2).

**Table 1.** Linear selection differential (*s*_*ij*_) on *Moricandia arvensis* traits.

	Spring				Summer					
	*s*_*ij*_	*SE*	*t*	*p*	*s*_*ij*_	*SE*	*t*	*p*	Interaction	*p*
Floral traits										
Floral diameter	**0.25**	**0.12**	**2.07**	**0.041**	**0.98**	**0.27**	**3.68**	**0.001**	**1.80**	**0.006**
Corolla tube length	0.09	0.13	0.70	0.489	**0.58**	**0.28**	**2.05**	**0.044**	0.72	0.110
Cyanidin content	−0.17	0.12	1.41	0.163	−0.37	0.29	1.27	0.207	−2.17	0.170
Kaempferol content	**−0.23**	**0.12**	**1.84**	**0.068**	**−0.62**	**0.28**	**2.20**	**0.031**	0.65	0.616
Brightness	0.09	0.13	0.75	0.453	0.17	0.29	0.59	0.557	0.13	0.688
Chroma	0.04	0.13	0.29	0.774	−0.22	0.29	0.76	0.452	−1.54	0.355
Chromatic contrast	−0.05	0.13	0.36	0.720	−0.27	0.29	0.94	0.351	−0.32	0.403
Achromatic contrast	0.05	0.13	0.44	0.665	0.20	0.29	0.67	0.504	0.26	0.495
Corolla shape component 1	−0.13	0.12	1.03	0.304	−0.23	0.29	0.80	0.428	−0.06	0.830
Corolla shape component 2	−0.05	0.13	0.42	0.679	0.29	0.29	0.99	0.325	0.31	0.280
Corolla shape component 3	−0.18	0.12	1.46	0.146	−0.09	0.29	0.31	0.756	0.13	0.654
Corolla shape component 4	−0.05	0.13	0.36	0.718	−0.27	0.29	0.92	0.359	−0.18	0.538
Leaf economics spectrum traits										
Specific leaf area	0.13	0.12	1.05	0.295	−0.43	0.29	1.49	0.140	**−0.64**	**0.050**
Leaf dry matter content	−0.21	0.12	1.66	0.100	**−0.89**	**0.27**	**3.28**	**0.002**	−0.14	0.757
Nitrogen content	**0.33**	**0.12**	**2.71**	**0.008**	−0.29	0.29	0.98	0.329	**−0.65**	**0.034**
Carbon to nitrogen content	**−0.40**	**0.12**	**3.33**	**0.001**	0.02	0.30	0.08	0.938	−0.54	**0.098**
Phosphorous content	0.11	0.13	0.88	0.382	−0.22	0.32	0.70	0.484	**−0.78**	**0.045**
Potassium content	0.00	0.13	0.05	0.957	**−0.56**	**0.31**	**1.78**	**0.079**	**−0.64**	**0.034**
Life history traits										
Plant height	**0.64**	**0.11**	**5.94**	**0.001**	**0.95**	**0.27**	**3.52**	**0.001**	0.07	0.788
Number of flowers	**0.47**	**0.12**	**4.03**	**0.001**	**1.67**	**0.22**	**7.71**	**0.001**	**18.94**	**0.000**
Plant size	**0.78**	**0.10**	**8.08**	**0.001**	**1.41**	**0.24**	**5.88**	**0.001**	**0.88**	**0.000**
Number of ovules	**0.47**	**0.12**	**4.04**	**0.001**	**0.55**	**0.30**	**1.81**	**0.075**	**0.51**	**0.013**

Significance of selection differential was found using linear models (*Equation 3* in the *Methods* section). Numbers in bold indicate significant effects. See Supplementary Table S2 and Supplementary Method S3 for trait definition.

**Table 2.** Linear selection differential (*β*_*2*_) on plasticity slopes.

	Model 1				Model 2			
	*β*_2_	*SE*	*t*	*p*	*β*_2_	*SE*	*t*	*p*
Floral trait								
Floral diameter	0.022	0.139	0.158	0.875	0.003	0.136	0.020	0.984
Corolla tube length	−0.012	0.139	−0.089	0.930	0.036	0.146	0.244	0.808
Cyanidin content	−0.165	0.137	−1.200	0.234	−0.034	0.239	−0.143	0.887
Kaempferol content	−0.023	0.139	−0.165	0.869	0.460	0.344	1.336	0.186
Brightness	0.050	0.139	0.359	0.721	0.005	0.138	0.036	0.971
Chroma	−0.121	0.138	−0.875	0.384	−0.154	0.143	−1.081	0.283
Chromatic contrast	**−0.309**	**0.134**	**−2.306**	**0.024**	**−0.899**	**0.304**	**−2.954**	**0.004**
Achromatic contrast	0.203	0.137	1.482	0.143	0.165	0.158	1.040	0.302
Corolla shape component 1	0.097	0.138	0.702	0.485	0.099	0.138	0.719	0.474
Corolla shape component 2	−0.048	0.139	−0.346	0.730	0.056	0.215	0.263	0.794
Corolla shape component 3	−0.094	0.138	−0.683	0.497	−0.040	0.140	−0.283	0.778
Corolla shape component 4	0.042	0.139	0.303	0.763	0.047	0.145	0.325	0.746
Leaf economics spectrum traits								
Specific leaf area	0.182	0.137	1.327	0.189	0.199	0.145	1.370	0.175
Leaf dry matter content	−0.024	0.139	−0.176	0.861	0.047	0.147	0.319	0.751
Nitrogen content	0.177	0.137	1.289	0.202	0.147	0.146	1.004	0.319
Carbon to nitrogent content	0.144	0.138	1.046	0.299	0.169	0.159	1.066	0.290
Phosphorous content	0.149	0.149	1.001	0.321	0.230	0.180	1.277	0.206
Potassium content	0.037	0.150	0.250	0.803	−0.022	0.150	0.147	0.864
Life history traits								
Plant height	0.200	0.137	1.462	0.148	**0.550**	**0.123**	**4.473**	**0.001**
Number of flowers	**−0.557**	**0.123**	**4.540**	**0.001**	**0.564**	**0.122**	**4.611**	**0.001**
Plant size	**0.639**	**0.117**	**5.453**	**0.001**	−0.207	0.164	−1.262	0.211
Number of ovules	0.165	0.137	1.198	0.235	0.184	0.130	1.415	0.161

Selection differential was calculated as the relationship between the slopes of the reaction norms and the total fitness of the genotypes. The reaction norm slopes were included in the analyses as absolute values and fitness as the total number of seeds produced by each genotype combining all environments. In Model 1, the slopes were tested without including intercepts to find the total selection acting on plasticity (*Equation 5* in the *Methods* section). In Model 2, the slopes were tested including the intercepts to find the selection acting on plasticity independent on the trait mean values (*Equation 6* in the *Methods* section). Numbers in bold indicate significant effects. See Supplementary Table S2 and Supplementary Method S3 for trait definition.

Contrasting with what we found for floral traits, plasticity moved five of the six LES traits toward their optima values since the direction of the within-environment selection differentials were concordant with the across-environment plastic differences in trait values (Figure 2B, Table 1). So, selection moved SLA, as well as nitrogen, phosphorous, and potassium contents to higher values in spring and lower values in summer, whereas the carbon–nitrogen ratio was moved to lower values in spring and higher values in summer (Figure 2B). Stabilizing selection was also found for two LES traits during spring: nitrogen content and potassium content in leaves (Supplementary Table S7). In addition, selection acted making steeper the slopes of the reaction norms of these traits (Figure 2B, Table 2).

We found that LH traits were under strong positive selection in the two seasons, a situation indirectly selecting against LH plasticity. In fact, the selection differential analysis suggests that plasticity was adaptive for only one LH trait, plant size (Figure 2C). Selection on this trait was significantly stronger during summer, when plants were larger (Figure 2C, Table 1). There was also a significant selection for increasing the magnitude of the slope of its reaction norm toward larger plants in summer (Figure 2C, Table 2). However, as shown above (Supplementary Table S5), plasticity was not significant for this LH traits, canceling the impact of selection.

### Determination of the selective agents affecting floral polyphenism

**Figure 3.**
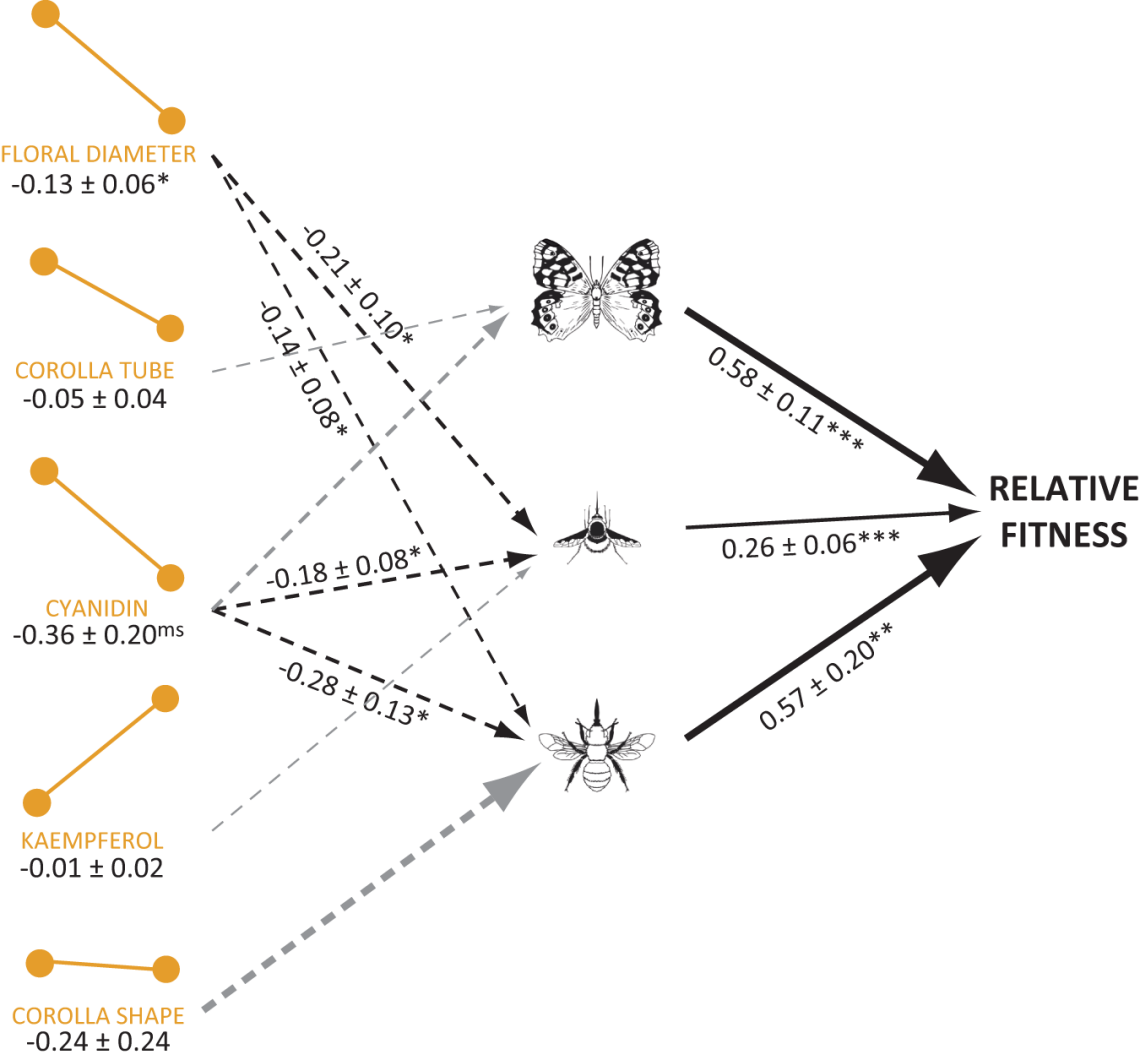
Selective scenario promoting the evolution of floral plasticity in *Moricandia arvensis*. Structural equation model relating relative fitness of the plants, the absolute values of the slopes of the reaction norms of floral traits, and the interaction strength of pollinators. Only three functional groups of pollinators (large butterflies, long-tongued large bees and beeflies) were kept in the final model. The model depicted is the definitive model obtained after an iterative process. The number below each trait is the total effect of that trait on fitness ± 1 *SE*. Dashed lines indicate negative relationships, whereas solid lines indicate positive relationships. Gray lines indicate nonsignificant relationships. Only significant path coefficients are shown (see Supplementary Table S8 for the overall statistical results). *p < 0.05, **p < 0.01, ***p < 0.001, ^ms^ marginally significant *p* < 0.1. Line widths are proportional to the magnitude of the effect of each connected variable. Pollinator icons were obtained from divulgare.net under a Creative Common licence.

Our definitive model adequately described the relationships between floral polyphenism, pollinators, and fitness (χ^2^ = 9.23, *p* = 0.683, df = 12, CFI = 1.000, RMSEA < 0.001 SRMR = 0.051, Supplementary Table S8). This model retained three pollinator groups, two visiting the flowers in both seasons (long-tongued large bees and large butterflies) and one visiting the flowers exclusively during spring (large beeflies). The model also retained the reaction norms of five floral traits (corolla diameter, corolla tube length, cyanidin content, kaempferol content, and the first component of corolla shape) (Figure 3). The relationships between the slopes of the floral reaction norms and the pollinators were always negative (Figure 3, Supplementary Table S8), mostly because pollinators prefer to visit those plants that displayed more similar flowers during spring and summer. So, long-tongued large bees preferred to visit plants bearing large flowers (spring: 0.02 ± 0.009, *p* = 0.09; summer: 0.04 ± 0.02, *p* = 0.02; within-season path coefficients ± 1 *SE*) with higher cyanidin content in summer (0.023 ± 0.039, *p* = 0.557) but lower in spring (−0.294 ± 0.103, *p* = 0.005). Large butterflies, although causing weaker impact due to their lower abundance, showed a similar pattern (Figure 3, Supplementary Table S8). So, they tended to visit plants with lower cyanidin content (−0.264 ± 0.104, *p* = 0.013) during spring and longer corolla tubes during summer (0.092 ± 0.097, *p* = 0.327). Finally, large beeflies, despite visiting the plants only during spring, visited more frequently those plants having smaller flowers (−0.026 ± 0.213, *p* = 0.903) with low cyanidin content (−0.155 ± 0.106, *p* = 0.146).

### Cost of floral plasticity

We found evidence of cost in only one floral trait, the chromatic contrast of the corolla, and only during summer (β_2_ = −1.98 ± 0.68, *t* = 2.93, *p* = 0.01; Supplementary Table S9). However, they must be taken cautiously because, in most cases, environment-specific trait values were correlated with plasticity (Supplementary Table S9).

### Factors mediating the expression of floral polyphenism

**Figure 4.**
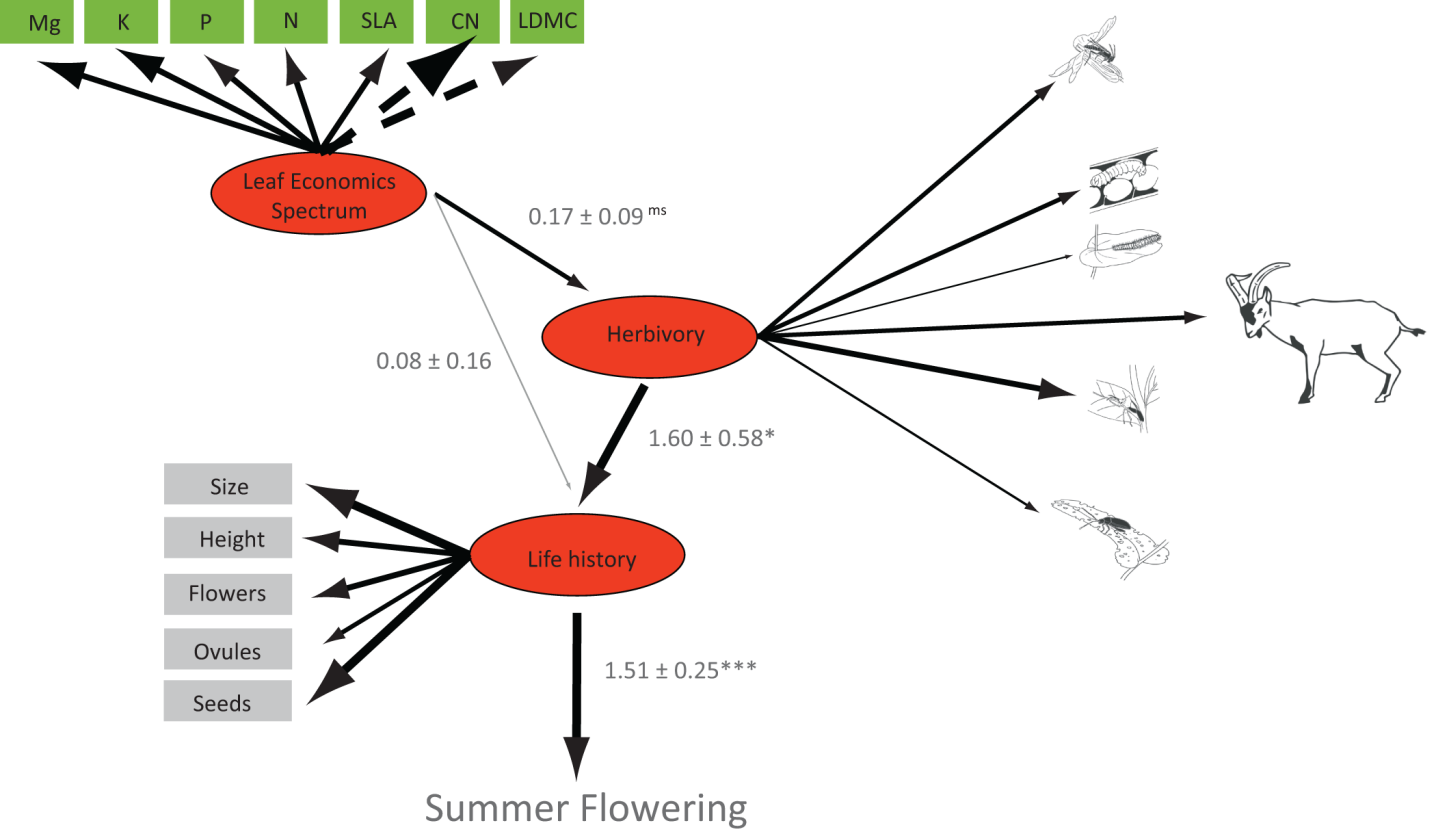
Factors shaping summer flowering. Structural equation model exploring the factors promoting summer flowering. Traits included in red ellipses were modeled as latent variables defined by the observed traits connected to them (see Table S10 for the overall statistical results). Dashed lines indicate negative relationships, whereas solid lines indicate positive relationships. Line widths are proportional to the magnitude of the effect of the latent variable on summer flowering or the magnitude of the relationship between traits and their associated latent variable. *p < 0.05, **p < 0.01, ***p < 0.001, ^ms^marginally significant *p* < 0.1. Herbivores are from top to bottom: nectar robbers, seed predators, butterfly chewers, ungulates, large sapsuckers, and leaf chewers. Animal icons were obtained from divulgare.net under a Creative Common licence.

Genetic similarity based on microsatellite loci did not appear to directly mediate the ability to flower during summer (exact G test χ^2^ = 15.54, df = 14, *p* = 0.342; *N* = 100 plants from Negratín population; Supplementary Figures S2 and S3). In contrast, the ability of plants to flower during summer was successfully described by the combined effect of the spring LH of plants, their resource-acquisitive strategy, and the stress caused by the impact of herbivores (χ^2^ = 162.70, *p* = 0.18, df = 147, CFI = 0.992, RMSEA = 0.033, SRMR = 0.098, Supplementary Table S10). The resulting structural model suggested that those plants growing larger and producing more flowers, ovules, and seeds during spring had more probability of flowering during summer (*R*^2^ = 0.94, total effect = 1.51 ± 0.25, *p* < 0.0001; Figure 4; Supplementary Table S10). The intensity of interaction with herbivores also influenced the probability of flowering during summer (total effect = 2.42 ± 0.91, *p* = 0.008). Six guilds of herbivores (nectar robbers, seed predators, leaf-eating pierid larvae, ungulates, foliar sapsuckers, and leaf-chewing beetles) boosted the size of the plants and augmented the number of spring flowers and seeds (*R*^2^ = 0.82, direct effect: 1.60 ± 0.58, *p* = 0.005), all of this indirectly resulting in a higher chance of summer flowering (Figure 4; Supplementary Table S10). Finally, LES traits also influenced the probability of flowering during summer (total effect = 0.53 ± 0.11, *p* < 0.0001). This effect was again indirect. Plants producing spring leaves with higher specific leaf area and foliar nitrogen concentration, lower carbon-to-nitrogen ratio, and thinner leaves were bigger and produced more seeds (0.08 ± 0.16, *p* = 0.61) and, above all, attracted more herbivores during spring (0.17 ± 0.09, *p* = 0.06) (Figure 4; Supplementary Table S10). By doing this, they increased their probability of flowering during summer and expressing floral polyphenism (Figure 4; Supplementary Table S10).

## Discussion

We have used three methods to explore the adaptive value of floral polyphenism in *M. arvensis*. According to the first method, which states that plasticity is adaptive when it implies a fitness gain, floral polyphenism appears to be adaptive as plastic individuals produced more seeds than nonplastic individual. Given that this species is mostly annual (Gómez et al., 2020), this increase in seed production implies an increase in the lifetime fitness of plastic individuals. Furthermore, our study also indicates that the cost of floral polyphenism is negligible in *M. arvensis*. This suggests that the benefits of floral plasticity outweigh their costs, a necessary condition for plasticity to evolve (Auld et al., 2010; Hoverman & Relyea, 2008; Scheiner & Levis, 2021; van Buskirk & Steiner, 2009). In fact, floral polyphenism seems to be an ancient trait in *M. arvensis* that has not been eliminated during its evolutionary history and it is currently expressed throughout its entire range (Gómez et al., 2020). In contrast, according to the second method, which proposes that plasticity is adaptive when selection increases the slopes of reaction norms, floral polyphenism does not appear to be adaptive. In fact, there was no evidence of selection acting to increase the slope of the reaction norm of any floral trait. This result suggests that natural selection does not favor plasticity (Arnold, Kiruuk, et al., 2019; Arnold, Nicotra, et al., 2019; Gotthard & Nylin, 1995; van Buskirk & Steiner, 2009). Therefore, the observed fitness advantages of floral polyphenism could be a consequence of a correlation with other traits rather than the direct action of selection (Via & Lande, 1985). The outcome of the second method agrees with that found using the third method, which states that plasticity is adaptive when selection moves the traits closer to optimal values within each environment. In fact, it appears that plasticity is moving the floral phenotype of *M. arvensis* away from its within-environment optimal values. This was most evident in the case of the summer floral morph. For example, selection favored large flowers in summer although plasticity caused flowers to be smaller in size in summer. Similarly, selection favored lower values of kaempferol content in summer when, again, plasticity caused flowers to produce more kaempferol in summer than in spring. Taken together, our results indicate that, despite its positive relationship with fitness, floral polyphenism does not appear to be adaptive in *M. arvensis* and suggests that determining the adaptive nature of plasticity requires not only quantifying its benefits on fitness but also a deeper understanding of the patterns of selection acting on plastic traits.

A probable explanation for the observed negative selection on *M. arvensis* floral polyphenism is the interaction with pollinators. The flowers of *M. arvensis* are visited by different sets of insects in spring and summer (Gómez et al., 2020). Whereas in spring, long-tongued large bees belonging to the Anthophorini subfamily were the main floral visitors, in summer, the flowers were visited mainly by short-tongued bees, flies, butterflies and, to a lesser extent, also by long-tongued large bees. Long-tongued large bees are much more effective as pollinators than any other functional group (Valverde et al., 2019). From the plant’s point of view, it is much more advantageous to attract these pollinators in both seasons. However, long-tongued large bees preferred to visit spring-morph flowers even when exposed to both types of flowers in common arenas (Gómez et al., 2020). This agrees with previous studies showing that these pollinators are the most frequent visitors of other *Moricandia* species as well as of other related Brassicaceae species displaying similar flowers to the spring-type flower of *M. arvensis*, such as *Rytidocarpus moricandioides*, *Raphanus* spp., or *Eruca* spp. (Barazani et al., 2019; Dukas & Shmida, 1989; Gómez, 1996; Gómez et al., 2015, 2016, 2022; González-Megías, 2016; Küchmeister et al., 1995; Shakeel et al., 2019). Due to this preference for spring-morph flowers, our structural equation model showed that long-tongued large bees exerted a significant selection against the plasticity of some floral traits, such as corolla shape and size. In addition, large butterflies and large beeflies, two pollinators visiting the flowers mostly during the summer, exerted a similarly significant selection against plasticity of certain floral traits, like corolla tube, corolla diameter, or floral pigments. Both spring and summer pollinators consistently selected against floral polyphenism in *M. arvensis*. Several recent studies have shown that plasticity can alter the patterns of selection imposed by pollinators (Dorey & Schiestl, 2022; Ramos & Schiestl, 2019). Our study shows for the first time that pollinators can directly impact the evolution of floral plasticity.

The maintenance of nonadaptive floral polyphenism in *M. arvensis* may be related to the fact that polyphenic plants are only those that flower in summer. And since summer flowering improves the lifetime fitness of the plants, selection is probably acting to increase the ability of the plants to continue flowering throughout the summer. We found that *M. arvensis* summer flowering was directly favored by a seasonal change in the expression of LES traits. This plasticity was adaptive for most LES traits, with plants producing optimal traits each season. *Moricandia arvensis* produced denser and thicker leaves with more structural carbon and higher water use efficiency during summer than during spring (Gómez et al., 2020). The LES describes strong relationships between multiple functional leaf traits that determine resource fluxes in vascular plants. Phenotypic plasticity of these traits is significant in many plants from water-limited environments and is positively associated with reproductive traits (Matesanz & Ramírez-Valiente, 2019; Sultan, 2000). In addition, LES traits also favored summer flowering indirectly by modulating the impact of some herbivores during spring. Plants displaying larger leaves with more nutrients this season attracted more herbivores. Attacked plants, rather than suffering a decrease in fitness, overcompensated against damage by growing more intensely during spring, a process that increased the probability of flowering during summer. Overcompensation against herbivores has been recorded in several plant species (Agrawal, 2000; García & Eubanks, 2018), including the genus *Moricandia* (Aguirrebengoa et al., 2021). Altogether, it seems that LES traits help plants flower during summer through two mechanisms, indirectly inducing overcompensation during spring and directly by exhibiting adaptive plasticity that allows summer leaves to be active and deliver resources to reproduction. A formal analysis of the strength of indirect LES-mediated selection on floral traits and plasticity would be desirable. Unfortunately, this analysis would require a much larger sample size, considering that there are many floral and nonfloral traits, and each trait is defined by at least two values, one per season.

Our study suggests that floral polyphenism in *M. arvensis* is probably a consequence of the inability of the plants to produce the spring floral morph when enduring hot and dry conditions. The absence of canalization in the *M. arvensis* flower contrasts with the widespread developmental canalization shown by the flowers of most Angiosperm species (Pélabon et al., 2011). Our study suggests that, because floral polyphenism is not adaptive in *M. arvensis*, floral canalization is surely limited due to the direct effects of the environment on the development of the flowers. For example, the shift in the color of the petals is caused by a concomitant shift in the pattern of expression of certain regulator genes that causes a modification of the anthocyanin biosynthetic pathway from cyanidins to flavonols (Gómez et al., 2020). Likewise, the decrease in the size of flowers during summer is probably a direct consequence of a lower photosynthetic rate and the depletion of water resources during the harsh summer (Flexas et al., 2014). It is remarkable that, despite potentially being the mere consequence of the environmental factors on the developmental pathway of the flower, the two floral morphs are highly integrated. This suggests that, although showing no macroenvironmental canalization, floral morphs of *M. arvensis* are highly canalized within each environment.

In brief, our study has revealed a complex scenario where a nonadaptive floral polyphenism has been maintained during the evolutionary history of *M. arvensis* as an indirect consequence of the benefit of extending flowering phenology and blooming during summer. Under these conditions, floral polyphenism seems to be a consequence of environmentally induced passive effects rather than active plasticity evolved to attract efficient pollinators. Floral polyphenism is thus mostly a byproduct of stressful summer conditions rather than an adaptation to summer pollinators. We postulate that nonadaptive plasticity of integrated and complex traits can evolve when there are limits to their canalization imposed by the environment and the expression of the traits is associated with a fitness benefit in that environment.

## Supplementary Material

qrae017_suppl_Supplementary_Materials

qrae017_suppl_Supplementary_Dataset_S1

qrae017_suppl_Supplementary_Dataset_S2

## Data Availability

All data used in this study have been included in the manuscript and in Supplementary Dataset S1 and S2.
