# Supplementary material for: Selection maintains a nonadaptive floral polyphenism
Source: Evol Lett. 2024 Apr 25;8(4):610–21. doi: 10.1093/evlett/qrae017 (PMC11291621; doi:10.1093/evlett/qrae017)
Supplement: qrae017_suppl_Supplementary_Materials [file qrae017_suppl_supplementary_materials.docx]

Article title: **Evolution of non-adaptive floral polyphenism**

Authors: José M. Gómez, Adela González-Megías, Cristina Armas, Eduardo Narbona, Luis Navarro, Francisco Perfectti

The following Supporting Information is available for this article:

**Dataset S1.** Phenotypic traits values and slopes for each plant used in the selection analyses.

**Dataset S2**. Microsatellite genotypes for 100 individuals.

**Figure S1**. Floral polyphenism in *M. arvensis*.

**Figure S2**. Genetic dissimilarity between *summer* individuals flowering and not flowering.

**Figure S3**. Genetic clustering of *M. arvensis* individuals.

**Table S1.** Proportion of plants flowering summer in five populations.

**Table S2.** List of the phenotypic traits considered in this study.

**Table S3**. Interaction between *M. arvensis* and each pollinator functional group.

**Table S4.** Interaction between *M. arvensis* and each herbivore guild.

**Table S5.** Outcome of the random regressions testing for occurrence of phenotypic plasticity.

**Table S6**. Fitness difference between *M. arvensis* plants flowering and not flowering in summer.

**Table S7.** Stabilizing selection differential on *M. arvensis* traits.

**Table S8.** Structural equation modelling testing the relationship between floral plasticity, pollinators and the fitness of *M. arvensis*.

**Table S9**. Costs of plasticity and cost of canalization of *M. arvensis* floral traits.

**Table S10.** Structural Equation Modelling determining the factors mediating summer flowering.

**Methods S1**. Plant phenotype assessment.

**Methods S2**. Statistical models.

**Methods S3.** Quantification of the interaction with pollinators and herbivores.

**Methods S4**. Genetic analyses.

**Methods S5.** Structural Equation Modelling determining the factors mediating summer flowering.

**Supplementary references.**

**Figure S1**. Floral polyphenism in *M. arvensis*. Spring (left) and summer (right) floral morphs.

**Figure S2.** Figure showing the location of the 100 plants of Negratín population in a two-dimensional space obtained after PCA from the genotypes for seven SSR loci. The plants that only flowered in spring are depicted in red. Two-first dimensions eigenvalues: 0.243, 0.202**.**

**Figure S3.** Dendrogram of the 100 plants of the Negratín population obtained using Nei-distances and UPGMA as clustering algorithm. Those plants that did not flower in summer appear in red. Please note that they are intermingled with those flowering both in spring and summer.

**Table S1.** Proportion of plants flowering in summer in five populations of the SE of Iberian Peninsula (100 plants per population). Average daily temperatures (°C) of each site are also indicated.

|  |  |  |  |  |  | **Average daily temperature (°C)** | |
| --- | --- | --- | --- | --- | --- | --- | --- |
| **Populations** | **Latitude** | **Longitude** | **Plants flowering during summer (%)** |  | **Plastic plants during summer (%)** | **Spring** | **Summer** |
| Negratín | 37º 33.7' N | 3º 57.5' W | 76 |  | 100 | 14.74 | 27.11 |
| Tabernas | 37° 00.3' N | 2° 27.4' W | 56 |  | 100 | 16.06 | 26.48 |
| Olula | 37° 23.3' N | 2° 17.9' W | 54 |  | 100 | 15.08 | 26.69 |
| Quesada | 37° 48.3' N | 3° 03.4' W | 44 |  | 100 | 15.54 | 27.92 |
| Malahá | 37° 08.4' N | 3° 43.9' W | 80 |  | 100 | 15.09 | 26.82 |

**Table S2.** List of the phenotypic traits considered in this study.

| **Trait** | **Type of trait** | **Definition** |
| --- | --- | --- |
| Corolla diameter | Floral | Distance between the edge of two opposite petals |
| Corolla tube length | Floral | Distance between corolla tube aperture and base of the sepals |
| Cyanidin content | Floral | Amount of anthocyanins in the corolla estimated as cyanidin-3-glucoside equivalents |
| Kaempferol content | Floral | Amount of flavonols in the corolla estimated as kaempferol-3-glucoside equivalents |
| Chromatic contrast | Floral | The distance of each petal colour loci to background in the colour hexagon model for bees |
| Achromatic contrast | Floral | The degree to which each petal colour loci generates an excitation value different from 0.5 in the green receptor using the colour hexagon model for bees |
| Brightness | Floral | The sum of the reflectance values over the entire reflectance spectrum |
| Chroma | Floral | The difference between the maximum and the minimum values of reflectance between the average reflectance of the spectrum |
| Corolla shape Component 1 | Floral | The 1^st^ component of the PCA geometric morphometric analysis of corolla shape |
| Corolla shape Component 2 | Floral | The 2^nd^ component of the PCA geometric morphometric analysis of corolla shape |
| Corolla shape Component 3 | Floral | The 3^rd^ component of the PCA geometric morphometric analysis of corolla shape |
| Corolla shape Component 4 | Floral | The 4^th^ component of the PCA geometric morphometric analysis of corolla shape |
| SLA | Leaf economics spectrum | Ratio between fresh leaf area and dry leaf mass |
| LDMC | Leaf economics spectrum | Ratio between dry leaf mass and fully rehydrated leaf mass |
| Nitrogen content | Leaf economics spectrum | Ratio of Nitrogen and dry leaf mass |
| CN ratio | Leaf economics spectrum | Ratio of Carbon to Nitrogen concentrations |
| Phosphorous content | Leaf economics spectrum | Ratio of Phosphorous and dry leaf mass |
| Potassium content | Leaf economics spectrum | Ratio of Potassium and dry leaf mass |
| Magnesium content | Leaf economics spectrum | Ratio of Magnesium and dry leaf mass |
| Number of flowers | Life history | Number of total flowers produced per plant |
| Plant height | Life history | Height of the tallest inflorescence |
| Plant size | Life history | Volume of the aerial part |
| Seed number | Life history | Number of seeds produced per plant |

**Table S3.** Intensity of interaction (estimated as number of insects per plant per 5 minutes) by each pollinator functional group visiting the flowers of *Moricandia arvensis* during spring and summer in Negratín population during 2018 and 2019.

| **Pollinator functional group** | **Spring** | **Summer** |
| --- | --- | --- |
| **Hymenoptera** |  |  |
| Short-tongued large bees | 0.430 ± 0.234 | 1.750 ± 0.612 |
| Short-tongued medium-sized bees | 0.360 ± 0.118 | 0.447 ± 0.172 |
| Short-tongued small bees | 1.700 ± 0.405 | 4.526 ± 0.943 |
| Short-tongued extra small bees | 0.080 ± 0.044 | 0.842 ± 0.171 |
| Long-tongued large bees | 13.160 ± 1.880 | 2.053 ± 0.492 |
| Pollen wasps | 0.020 ± 0.014 | - |
| Large wasps | - | 0.026 ± 0.018 |
| Small wasps | 0.280 ± 0.127 | 0.039 ± 0.022 |
| Small ants | - | 0.013 ± 0.013 |
| Large ants | 0.190 ± 0.063 | 0.816 ± 0.252 |
| **Lepidoptera** |  |  |
| Large butterflies | 0.310 ± 0.205 | 0.355 ± 0.143 |
| Small butterflies | - | 0.026 ± 0.018 |
| Hawkmoths | 0.160 ± 0.115 | - |
| **Diptera** |  |  |
| Large beeflies | 1.340 ± 0.397 | - |
| Small beeflies | - | 0.053 ± 0.037 |
| Large hoverflies | 0.010 ± 0.010 | 0.013 ± 0.013 |
| Small hoverflies | 0.020 ± 0.020 | 0.395 ± 0.123 |
| Large flies | 0.010 ± 0.010 | 0.066 ± 0.054 |
| Long-tongued flies | - | 0.026 ± 0.018 |
| Small flies | 0.001 ± 0.001 | 0.026 ± 0.018 |
| **Coleoptera** |  |  |
| Large beetles | 0.660 ± 0.179 | 0.224 ± 0.098 |
| Small beetles | 0.150 ± 0.050 | 0.973 ± 0.112 |
| Small diving beetles | 1.470 ± 0.410 | 0.145 ± 0.045 |
| **Other Orders** |  |  |
| Thrips | - | 0.053 ± 0.026 |
| Aphids | - | 0.013 ± 0.013 |
| Bugs | 0.010 ± 0.010 | 0.184 ± 0.052 |

**Table S4.** Intensity of interaction by each herbivore guild attacking *Moricandia arvensis* during spring and summer in Negratín population.

| **Herbivore guild** | **Unit of interaction** | **Spring** | **Summer** |
| --- | --- | --- | --- |
| **Leaves** |  |  |  |
| Butterfly chewers | Number of individuals | 0.42 ± 0.18 | - |
| Leaf chewers (*Phyllotreta*) | Percentage of leaves attacked | 1.22 ± 0.29 | 2.80 ± 0.48 |
| Other beetles | Number of individuals | 0.15 ± 0.04 | 0.03 ± 0.02 |
| Leaf miners | Percentage of leaves attacked | 0.14 ± 0.05 | - |
| Large sapsuckers | Number of individuals | 0.14 ± 0.05 | - |
| Small sapsuckers | Percentage of leaves attacked | 0.14 ± 0.07 | 1.95 ± 1.07 |
| **Fruits & Seeds** |  |  |  |
| Galls | Percentage of fruits attacked | 0.04 ± 0.04 | - |
| Avian seed predators | Percentage of fruits consumed | 2.69 ± 0.62 | 17.57 ± 3.12 |
| Insect seed predators | Percentage of seeds consumed | 3.46 ± 0.75 | 5.52 ± 2.36 |
| **Flowers & Inflorescences** |  |  |  |
| Large sapsuckers | Number of individuals | 0.31 ± 0.09 | 0.50 ± 0.10 |
| Small sapsuckers | Percentage of inflorescences attacked | 1.90 ± 0.56 | 4.53 ± 1.64 |
| Butterfly larvae | Number of individuals | 0.23 ± 0.63 | 0.17 ± 0.53 |
| Folivorous beetles | Number of individuals | 0.65 ± 1.29 | 0.03 ± 0.16 |
| Floral chewers | Percentage of floral buds consumed | 1.95 ± 0.60 | 4.21 ± 1.39 |
| Ungulates | Percentage of inflorescences consumed | 14.20 ± 1.07 | - |
| Nectar robbers | Percentage of flowers robbed | 7.79 ± 0.99 | 4.38 ± 9.74 |
| Galls | Percentage of flowers attacked | 0.01 ± 0.07 | 0.03 ± 0.23 |

**Table S5.** Outcome of the random regressions testing for occurrence of phenotypic plasticity in all traits from the plants inhabiting the studied populations. *P-values* are the significance of the population-wide reaction-norm slope (E), the differences in trait values among individuals (G) and the among-individual differences in individual reaction-norm slopes.

|  | **E** | **G** | **E x G** |
| --- | --- | --- | --- |
| **Floral traits** |  |  |  |
| Floral diameter | 0.02 | 0.00001 | 0.00001 |
| Corolla tube length | 0.00001 | 0.001 | 0.00001 |
| Cyanidin content | 0.00001 | 0.00001 | 0.00001 |
| Kaempferol content | 0.05 | 0.00001 | 0.00001 |
| Brightness | 0.05 | 0.00001 | 0.00001 |
| Chroma | 0.00001 | 0.00001 | 0.00001 |
| Chromatic contrast | 0.00001 | 0.00001 | 0.00001 |
| Achromatic contrast | 0.02 | 0.00001 | 0.00001 |
| Corolla shape component 1 | 0.99 | 0.0001 | 0.00001 |
| Corolla shape component 2 | 0.00001 | 0.0001 | 0.00001 |
| Corolla shape component 3 | 0.81 | 0.06 | 0.002 |
| Corolla shape component 4 | 0.00001 | 0.00001 | 0.004 |
| **Leaf economics spectrum traits** |  |  |  |
| Specific leaf area | 0.00001 | 0.00001 | 0.00001 |
| Leaf dry matter content | 0.00001 | 0.00001 | 0.00001 |
| Nitrogen content | 0.00001 | 0.00001 | 0.00001 |
| Carbon to nitrogen content | 0.00001 | 0.00001 | 0.00001 |
| Phosphorous content | 0.00001 | 0.00001 | 0.00001 |
| Potassium content | 0.00001 | 0.00001 | 0.00001 |
| **Life history traits** |  |  |  |
| Plant height | 0.12 | 0.00001 | 0.00001 |
| Number of flowers | 0.44 | 0.00001 | 0.00001 |
| Plant size | 0.80 | 0.00001 | 0.00001 |
| Number of ovules | 0.02 | 0.00001 | 0.00001 |

**Table S6**. Fitness difference between *Moricandia arvensis* plants flowering (76) and not flowering (24) in summer. One-way ANOVA, df=1,98. For ovules per fruit and seeds per fruit in summer, we compared both spring and summer fruits of summer-flowering plants against spring fruits of plants that did not flower during summer and produced flowers only during spring.

| **Fitness component** | **SS** | **F** | **P** |
| --- | --- | --- | --- |
| Fruits | 8.78 | 9.54 | 0.003 |
| Ovules per fruit - SPRING | 5.85 | 6.16 | 0.015 |
| Ovules per fruit - SUMMER | 2.14 | 2.17 | 0.144 |
| Seeds per fruit - SPRING | 1.71 | 1.72 | 0.192 |
| Seeds per fruit - SUMMER | 29.3 | 41.2 | 0.001 |
| Seeds per plant | 6.53 | 6.92 | 0.010 |

**Table S7.** Stabilizing selection differential (ɣ) on *M. arvensis* traits. We estimated stabilizing selection by means of a quadratic linear model (equation 3 in methods). Numbers in bold indicate significant effects suggesting stabilizing selection (i.e. only those significant values associated with negative quadratic estimates). See methods for units of measure and the definition of all floral traits.

|  | Spring | | | |  | Summer | | | |
| --- | --- | --- | --- | --- | --- | --- | --- | --- | --- |
|  | ɣ | SE | t | p |  | ɣ | SE | t | p |
| **Floral traits** |  |  |  |  |  |  |  |  |  |
| Floral diameter | -1.39 | 1.22 | -1.14 | 0.26 |  | 3.02 | 2.45 | 1.24 | 0.22 |
| Corolla tube length | -0.60 | 1.25 | -0.48 | 0.63 |  | -0.81 | 2.61 | -0.31 | 0.76 |
| Cyanidin content | 1.30 | 1.23 | 1.05 | 0.30 |  | 2.78 | 2.62 | 1.06 | 0.29 |
| Kaempferol content | -0.28 | 1.23 | -0.23 | 0.82 |  | 2.54 | 2.57 | 0.99 | 0.33 |
| Brightness | 1.20 | 1.24 | 0.96 | 0.34 |  | -2.54 | 2.66 | -0.96 | 0.34 |
| Chroma | 0.41 | 1.25 | 0.33 | 0.75 |  | -4.37 | 2.62 | -1.67 | 0.10 |
| Chromatic contrast | 1.06 | 1.25 | 0.85 | 0.40 |  | -4.54 | 2.60 | -1.74 | 0.09 |
| Achromatic contrast bees | 1.39 | 1.24 | 1.12 | 0.27 |  | -1.96 | 2.52 | -0.78 | 0.44 |
| Corolla shape component 1 | 3.91 | 1.18 | 3.32 | 0.00 |  | -3.28 | 2.65 | -1.24 | 0.22 |
| Corolla shape component 2 | -1.51 | 1.24 | -1.22 | 0.23 |  | 2.09 | 2.64 | 0.79 | 0.43 |
| Corolla shape component 3 | 0.49 | 1.24 | 0.39 | 0.70 |  | -2.11 | 2.67 | -0.79 | 0.43 |
| Corolla shape component 4 | 0.42 | 1.25 | 0.33 | 0.74 |  | -1.17 | 2.67 | -0.44 | 0.66 |
|  |  |  |  |  |  |  |  |  |  |
| **Leaf economics spectrum traits** |  |  |  |  |  |  |  |  |  |
| Specific leaf area | -1.95 | 1.23 | -1.59 | 0.12 |  | 1.34 | 2.62 | 0.51 | 0.61 |
| Leaf dry matter content | -0.90 | 1.23 | -0.73 | 0.47 |  | 1.46 | 2.32 | 0.63 | 0.53 |
| Nitrogen content | **-3.28** | 1.16 | -2.83 | 0.01 |  | 0.79 | 2.66 | 0.30 | 0.77 |
| Carbon to nitrogen content | 1.76 | 1.17 | 1.50 | 0.14 |  | -1.84 | 2.67 | -0.69 | 0.49 |
| Phosphorous content | **-2.82** | 1.22 | -2.30 | 0.02 |  | 1.96 | 2.66 | 0.74 | 0.46 |
| Potassium content | -1.44 | 1.25 | -1.15 | 0.25 |  | 1.18 | 2.62 | 0.45 | 0.65 |
|  |  |  |  |  |  |  |  |  |  |
| **Life history traits** |  |  |  |  |  |  |  |  |  |
| Plant height | 2.76 | 1.04 | 2.67 | 0.01 |  | 1.21 | 2.46 | 0.49 | 0.63 |
| Number of flowers | **-3.53** | 1.10 | -3.20 | 0.01 |  | **-4.13** | 1.84 | -2.25 | 0.03 |
| Plant size | **-3.11** | 0.92 | -3.40 | 0.01 |  | **4.48** | 2.12 | 2.11 | 0.04 |
| Number of ovules | -1.02 | 1.15 | -0.88 | 0.38 |  | -2.35 | 2.60 | -0.91 | 0.37 |

**Table S8.** Outcome of the structural equation modelling testing the relationship between floral plasticity, pollinators and the fitness of *M. arvensis*. The dependent variable has been modeled as continuous, the estimator of the model has been ML, and the optimization method NLMINB.

|  | Variables | Estimate | SE | z value | P value |
| --- | --- | --- | --- | --- | --- |
| **Regressions** |  |  |  |  |  |
| Long-tongued large bees | Lifetime relative fitness | 0.58 | 0.20 | 2.91 | 0.004 |
| Large beeflies | Lifetime relative fitness | 0.26 | 0.06 | 4.62 | 0.001 |
| Large butterflies | Lifetime relative fitness | 0.58 | 0.11 | 5.49 | 0.001 |
| Floral diameter | Long-tongued large bees | -0.13 | 0.08 | -1.74 | 0.082 |
| Cyanidin content | Long-tongued large bees | -0.27 | 0.13 | -2.18 | 0.030 |
| Corolla shape component 1 | Long-tongued large bees | -0.42 | 0.45 | -0.92 | 0.358 |
| Floral diameter | Large beeflies | -0.20 | 0.10 | -2.09 | 0.036 |
| Kaempferol content | Large beeflies | -0.03 | 0.10 | -0.31 | 0.759 |
| Cyanidin content | Large beeflies | -0.18 | 0.08 | -2.11 | 0.035 |
| Corolla Tube | Large butterflies | -0.09 | 0.07 | -1.28 | 0.201 |
| Cyanidin content | Large butterflies | -0.27 | 0.23 | -1.19 | 0.235 |
| **Total Effects** | |  |  |  |  |
| Floral diameter |  | -0.13 | 0.06 | -2.28 | 0.023 |
| Corolla tube |  | -0.05 | 0.04 | -1.17 | 0.242 |
| Cyanidin content |  | -0.36 | 0.20 | -1.84 | 0.060 |
| Kaempferol content |  | -0.01 | 0.03 | -0.30 | 0.763 |
| Corolla shape component 1 |  | -0.24 | 0.24 | -1.00 | 0.316 |

**Table S9**. Costs of plasticity and cost of canalization of *M. arvensis* floral traits. We used the model proposed by Scheiner & Berrigan (1998) in which the cost is estimated as the slope (β_2_) between environment-specific fitness and trait plasticity (see equation 7 in Methods S2). We also show the environment-specific correlations between trait values and plasticity, following the model proposed Auld *et al.* (2010).

|  | **Spring** | | | | | | |  |  | **Summer** | | | | | |
| --- | --- | --- | --- | --- | --- | --- | --- | --- | --- | --- | --- | --- | --- | --- | --- |
|  | Cost | | | |  | Correlation | |  | Cost | | | |  | Correlation | |
| **Traits** | β_2_ | SE | t | p |  | r | p |  | β_2_ | SE | t | p |  | r | p |
| Floral diameter | -0.31 | 1.21 | -0.25 | 0.31 |  | **0.64** | **0.01** |  | 0.49 | 1.93 | 0.26 | 0.80 |  | **-0.58** | **0.01** |
| Corolla tube length | -0.06 | 0.82 | -0.07 | 0.06 |  | **0.56** | **0.01** |  | 1.96 | 2.00 | 0.98 | 0.33 |  | **-0.80** | **0.01** |
| Cyanidin content | -0.28 | 2.18 | -0.13 | 0.28 |  | **0.95** | **0.01** |  | -2.45 | 1.22 | -2.01 | 0.05 |  | **-0.29** | **0.01** |
| Kaempferol content | 0.34 | 0.49 | 0.70 | 0.34 |  | **0.11** | **0.01** |  | 2.27 | 3.98 | 0.57 | 0.57 |  | **0.98** | **0.01** |
| Brightness | 0.11 | 0.72 | 0.15 | 0.88 |  | 0.07 | 0.53 |  | 0.11 | 0.72 | 0.15 | 0.88 |  | 0.20 | 0.08 |
| Chroma | -2.46 | 1.98 | -1.25 | 2.46 |  | **-0.81** | **0.01** |  | -0.88 | 2.82 | -0.31 | 0.76 |  | **0.65** | **0.01** |
| Chromatic contrast* | -0.63 | 0.39 | -1.62 | 0.63 |  | 0.01 | 0.81 |  | **-1.98** | **0.68** | **-2.93** | **0.01** |  | 0.03 | 0.81 |
| Achromatic contrast | 0.57 | 0.51 | 1.13 | 0.57 |  | -0.10 | 0.92 |  | 1.67 | 0.91 | 1.84 | 0.07 |  | -0.01 | 0.92 |
| Corolla shape component 1 | 0.74 | 0.79 | 0.94 | 0.74 |  | -0.01 | 0.93 |  | -0.43 | 1.45 | -0.30 | 0.77 |  | 0.01 | 0.93 |
| Corolla shape component 2 | -0.39 | 1.11 | -0.35 | 0.39 |  | **-0.26** | **0.01** |  | 0.95 | 2.85 | 0.33 | 0.74 |  | **-0.73** | **0.01** |
| Corolla shape component 3 | -0.51 | 0.97 | -0.53 | 0.51 |  | **0.07** | **0.03** |  | -0.73 | 1.86 | -0.39 | 0.70 |  | **0.26** | **0.03** |
| Corolla shape component 4 | 0.27 | 1.11 | 0.24 | 0.27 |  | **-0.20** | **0.01** |  | 1.67 | 2.41 | 0.69 | 0.49 |  | **0.58** | **0.01** |

* We tested for additional cost of plasticity using Equation 8 in Methods S2. The result of the analysis suggests that there is not significant additional cost (β_3_ ± 1 SE = 1.05 ± 0.78, t= 1.350, p = 0.181).

**Table S10.** Outcome of the structural equation modelling testing the factors influencing the probability of flowering during summer. The dependent variable has been modeled as binomial, the estimator of the model has been DWLS, and the optimization method NLMINB.

|  | Variables | Estimate | Std.Err | z value | P value |
| --- | --- | --- | --- | --- | --- |
| **Latent** |  |  |  |  |  |
| Leaf Economics Spectrum | Specific leaf area | 1.00 | 0.68 | 0.69 |  |
| Leaf Economics Spectrum | Nitrogen content | 1.21 | 0.19 | 6.37 | 0.01 |
| Leaf Economics Spectrum | Leaf dry matter content | -0.92 | 0.15 | -6.06 | 0.01 |
| Leaf Economics Spectrum | Carbon to Nitrogent ratio | -1.40 | 0.25 | -5.61 | 0.01 |
| Leaf Economics Spectrum | Phosphorus content | 0.71 | 0.14 | 5.06 | 0.01 |
| Leaf Economics Spectrum | Potasium content | 0.29 | 0.15 | 1.91 | 0.06 |
| Leaf Economics Spectrum | Magnesium content | 0.33 | 0.14 | 2.43 | 0.02 |
| Herbivory | Ungulates | 1.00 | 0.35 | 0.35 |  |
| Herbivory | Seed predators | 0.74 | 0.26 | 2.88 | 0.01 |
| Herbivory | Leaf chewers | 0.51 | 0.30 | 1.71 | 0.09 |
| Herbivory | Nectar robbers | 0.62 | 0.30 | 2.08 | 0.04 |
| Herbivory | Leaf sapsuckers | 1.56 | 0.35 | 4.45 | 0.01 |
| Herbivory | Butterfly chewers | 0.29 | 0.27 | 1.06 | 0.29 |
| Life History | Number of flowers | 1.00 | 0.64 | 0.65 |  |
| Life History | Plant Size | 1.20 | 0.11 | 11.31 | 0.01 |
| Life History | Number of seeds | 1.12 | 0.16 | 7.16 | 0.01 |
| Life History | Plant height | 0.99 | 0.15 | 6.66 | 0.01 |
| Life History | Number of ovules | 0.65 | 0.13 | 5.13 | 0.01 |
| **Regressions** |  |  |  |  |  |
| Summer Flowering | Life History | 1.51 | 0.25 | 5.93 | 0.01 |
| Life History | Herbivory | 1.60 | 0.58 | 2.78 | 0.01 |
| Life History | Leaf Economics Spectrum | 0.08 | 0.16 | 0.51 | 0.61 |
| Herbivory | Leaf Economics Spectrum | 0.17 | 0.09 | 1.84 | 0.06 |
| **Total Effects** | |  |  |  |  |
| Summer Flowering | Life History | 1.53 | 0.25 | 6.03 | 0.01 |
| Summer Flowering | Herbivory | 2.42 | 0.91 | 2.44 | 0.01 |
| Summer Flowering | Leaf Economics Spectrum | 0.53 | 0.11 | 4.08 | 0.01 |

**Methods S1. Plant phenotype assessment**

**Floral traits**. We marked 100 co-occurring plants in the Negratín population (Table S1). In each of these plants, we measured, both during spring and during summer, corolla size, corolla pigments, corolla colour, and corolla shape.

Corolla size was estimated of each studied flower by mean of two traits: (1) Corolla diameter, estimated as the distance in mm between the edge of two opposite petals; (2) Corolla tube length, the distance in mm between the corolla tube aperture and the base of the sepals. These variables were measured using a digital calliper with ± 0.1 mm of error.

The colour pigments present in the corolla of *M. arvensis* are flavonoids, particularly anthocyanins (cyanidin derivatives) and flavonols (kaempferol derivatives) (Gómez *et al.* 2020). Colour pigments were estimated as (3) cyanidin content, expressed as cyanidin-3-glucoside equivalents in fresh weight (mg g^-1^ FW) and (4) kaempferol content, expressed as kaempferol-3-glucoside equivalents in fresh weight (mg g^-1^ FW). We quantified both types of pigment spectrophotometrically. In each plant, the corollas of two flowers of each colour morph were analysed. Briefly, flavonoids were extracted in 1.5 ml of MeOH:HCl (99:1% v:v) and stored at -80º C in the dark (Gómez *et al.* 2020). Two replicas of 200 μL for each sample were measured in a Multiskan GO microplate spectrophotometer (Thermo Fisher Scientific Inc., MA, USA), and total anthocyanins and flavonols were quantified as absorbance at 520 and 350 nm, respectively.

Corolla colour of the flowers was assessed by measuring their UV-visible spectral reflectance (300 to 700 nm). A petal of two flowers of each colour morph (N = 100) was measured using a FLAME spectrophotometer (Ocean Insight Inc., Orlando, USA) equipped with a deuterium–tungsten light source (200–2000 nm) and a probe holder to make measurements at 45° angle from the sample. The spectrophotometer was calibrated before measurements using a WS-1-SL white standard and the software Ocean View 2.0. We set 2 s of integration time and 12 boxcar width to maximize the amount of light used in reflectance measurements and to reduce occasionally erratic reflectance values at individual nm, respectively. Petal colour was estimated using four parameters; two parameters take into account the visual system of bees, and the other two are general for any group of pollinators: (5) Colour contrast, the distance of each petal colour loci to the background in the colour hexagon model for bees (i.e., chromatic contrast measured in hexagon units; Spaethe *et al.*, 2001). (6) Achromatic contrast, the excitation generated in the green photoreceptor of bees after adaptation to the background (i.e., green contrast measured in hexagon units; Spaethe *et al.*, 2001). Chromatic and achromatic contrasts provide behaviourally meaningful measures for flower conspicuousness to bees (Van der Kooi & Spaethe, 2022). These two indices are calculated for the bee visual system, given that the main pollinators of both flower colour morphs of *M. arvensis* are bees (Gómez *et al.*, 2020). We used the colour hexagon model for honeybees (Chittka *et al.*, 1994), but they work well for other groups of pollinating hymenopterans because their colour vision system is very similar (Briscoe & Chittka, 2001). Bees use colour and achromatic contrast to detect flowers at short and at long distances, respectively (Giurfa *et al.*, 1997). In spring, plants grew in an environment dominated by herbaceous species; thereby, we used the typical green leaves background (provided in Chittka *et al.*, 1994). In summer, plants were growing with virtually no surrounding vegetation, and we considered the bare soil as the background using the average reflectance of 10 soil samples. (7) Brightness, the sum of the reflectance values over the entire reflectance spectrum, represents an achromatic value of reflection of the sample. (8) Chroma, the difference between the maximum and the minimum values of reflectance between the average reflectance of the spectrum, is a measure of spectral purity. Brightness and chroma are particularly suitable for detecting broad trends in reflectance spectra and are independent of the pollinator's visual system (Endler & Mielke, 2005).

Corolla shape variation was studied using geometric morphometric tools based on a landmark-based methodology (Zelditch *et al.*, 2012). For this, we took a digital photo of the front view and planar position in each of the two selected flowers per individual plant studied in each of the four populations. We defined 32 co-planar landmarks covering the corolla shape and using midrib, primary and secondary veins and petal extremes and connections. From the two-dimensional coordinates of landmarks, we extracted shape information and computed the generalized orthogonal least-squares Procrustes averages using the Generalized Procrustes Analysis (GPA) superposition method. Due to the intrinsic symmetry pattern exhibited by Brassicaceae flowers, we did the analyses considering both the symmetric and asymmetric components of the shape (Savriama *et al.*, 2012; Schlager, 2017; Savriama 2018). We performed a Principal Component Analysis (PCA) on the GPA-aligned specimens, and afterwards, we did a Canonical Variate Analysis (CVA) to explore the difference in shape between seasons and populations (Zelditch *et al.*, 2012; Schlager, 2017). Corolla shape was afterwards described as (9-12) Corolla shape components 1 - 4, the four first principal components of the PCA. Geometric morphometric analyses were performed in the R packages ‘geomorph’ (Adams *et al.*, 2013), ‘Morpho’ (Schlager, 2017) and ‘shapes’ (Dryden & Mardia, 2016; Dryden, 2018).

**Leaf Economics Spectrum traits**. For the 100 co-occurring plants marked in the Negratín population, we measured, both in spring and summer, a set of leaf traits related to the leaf economics spectrum (Wright *et al.*, 2004) of the plants: (1) Specific leaf area (SLA, m^2^ kg^-1^) and (2) Leaf dry matter content (LDMC, mg g^-1^) were measured following standard protocols (Pérez-Harguindeguy *et al.*, 2016). We collected three fully expanded and mature leaves without any visible damage (e.g., herbivory, pathogen attack) from the base, midsection and apical part of outer stems (that is, leaves were not shaded by other leaves) and at random canopy aspects. Leaves were rehydrated overnight in the dark and subsequently weighted and scanned. Leaf area was measured using the Midebmp software (Almería, Spain). Leaves were dried in the oven at 60°C and weighed after 72 hours. From these measurements, we calculated the SLA as the one-sided area of the fully rehydrated fresh leaf divided by its dry mass, and the LDMC as the ratio between the leaf dry mass and the fully rehydrated fresh mass.

Leaf (3) Nitrogen content (N) and (4) Carbon-to-Nitrogen content (C:N ratio) were analysed in a couple of fully expanded, non-damaged leaves per plant. Oven-dry leaves were ground in a ball mill MM400 (Retsch GmbH, Haan, Germany) at 3000 rpm for 1 minute to obtain a fine powder, which was stored in Eppendorf tubes. We wrapped 0.003 g of each sample in tin capsules D1008 (Elemental Microanalysis, United Kingdom). Leaf C and N relative content (in mass percentage) were determined at the Laboratory of Stable Isotopes at the Estación Biológica de Doñana (EBD-CSIC) (http://www.ebd.csic.es/lie/index.html). All samples were combusted at 1020°C using a continuous flow isotope-ratio mass spectrometry system, by means of a Flash HT Plus elemental analyser coupled to a Delta-V Advantage isotope-ratio mass spectrometer via a CONFLO IV interface (ThermoFisher Scientific, Bremen, Germany). Leaf (5) Phosphorous content (P), (6) Potassium content (K), and (7) Magnesium content (Mg) were determined after acid digestion and with an inductively coupled plasma (ICP) emission spectrometer (ICAP 6500 DUO Thermo; Thermo Scientific, Wilmington, DE, USA) at the CSIC-CEBAS (Centre of Edaphology and Applied Biology of Segura) “Ionomics” laboratory in Murcia, Spain.

**Life history traits**. For the 100 co-occurring plants marked in the Negratín population, we measured, at the end of both in spring and summer, the following life history traits: (1) Plant height, the distance to the top of the tallest inflorescence each season. (2) Number of flowers. We counted the total number of flowers produced per individual plant both during spring and during summer. (3) Plant size, inferred per season by measuring the length and width of each plant and calculating the hemisphere as ½ * 4/3 * p * height * width * length. (4) Number of ovules and (5) Number of seeds, a lifetime estimate of reproductive plant fitness in this plant species (Gómez *et al.*, 2020). At the end of each season, we counted the proportion of flowers setting fruits and haphazardly collected 10 fruits per plant per season. Fruits were taken to the laboratory, where we opened the fruits and counted the total number of ovules per fruit and the proportion of them setting seeds. By multiplying the number of flowers by the ratio of fruits/flowers and seeds/fruits, we obtained the total number of seeds produced per plant each season. We used the same approach to obtain the number of ovules per plant and season.

**Methods S2. Statistical models**

**Within-individual plasticity.** The magnitude and significance of the within-individual plasticity were calculated for each phenotypic trait by random slope mixed models fitted as (Arnold *et al*., 2019):

| $z_{ij}=\alpha+\beta*e_{j}+a_{i}+b_{i}*e_{j}+\varepsilon_{ij}$ | [1] |
| --- | --- |

where *z_ij_* is the phenotypic trait value of genotype *i* on environment *j*; *e_j_* is the value of the environment experienced on occasion *j* (the mean-centred average daily temperature); α is the fixed effects of the overall intercept and describes the population mean trait value; β is the fixed effects of the overall slope regression coefficients and describes the population mean reaction norm slope; *a_i_* is the random intercept coefficient for individual *i* that represents the difference in means between individuals; *b_i_* is the random coefficient for individual *i* that represents the difference in slopes between individuals; and ε_ij_, the residual for individual *i* on occasion *j*.

**Within-environment selection differentials.** Directional selection differentials in each environment *j* (*s_j_*) were estimated by the covariance between that trait of each genotype *i* in that environment (*z_ij_*) and the genotypic values of environment-specific relative fitness (*w_ij_*) (Rice, 2004):

| *s_j_* = cov(*w_ij_*, *z_ij_*) | (2) |
| --- | --- |

Fitness was in this model environment-specific. The statistical significance of the selection differentials was calculated by fitting univariate linear models (Lande & Arnold, 1983; Lynch & Walsh, 1998).

| $w_{ij}=\alpha+\beta*z_{ij}+\varepsilon_{ij}$ | (3) |
| --- | --- |

Within-environment stabilizing selection was calculated by fitting univariate quadratic models as (Lande & Arnold, 1983):

| $w_{ij}=\alpha+\beta*z_{ij}+\frac{\gamma}{2}*z_{ij}^{2}+\varepsilon_{ij}$ | (4) |
| --- | --- |

When β = 0 and γ is significantly negative, the selection gradient indicates stabilizing selection (Lande & Arnold, 1983; Lynch & Walsh, 1998).

**Selection on the slopes of the reaction norms.** We performed a simple linear model relating the total relative fitness of each genotype *i* combining all environments ($w_{i\cdot}$) with the slope of each plastic trait. Total relative fitness was calculated as the sum of seeds produced by each genotype in each environment. The slope of the reaction norm of each genotype was obtained by performing random regression mixed models as explained in equation [1] and using the BLUP slopes as an estimate of the deviance of the plasticity of each genotype from the population-level plasticity (Arnold *et al*., 2019).

| $w_{i\cdot}=\alpha+\beta*slope(z_{i})+\varepsilon_{ij}$ | (5) |
| --- | --- |

Because the slope of the reaction norm is correlated with the average values of the trait for most plastic traits (Arnold *et al*., 2019), we performed a second multivariate mixed model including not only the slope but also the average value of the trait (${\overset{^}{z}}_{i}$) (van Kleunen & Fischer, 2005; Stinchcombe *et al*., 2004):

| $w_{i}=\alpha+\beta_{1}*{\overset{^}{z}}_{i}+\beta_{2}*slope\left( z_{i} \right)+\varepsilon_{ij}$ | (6) |
| --- | --- |

The average value of each trait was calculated as the individual BLUP intercepts of the random regression mixed models, as explained in equation [1].

**Cost of plasticity.** Maintenance and production costs of plasticity were estimated using the model proposed by Scheiner & Berrigan (1998):

| $w_{ij}=\alpha+{\beta_{1}*z}_{ij}+\beta_{2}*slope\left( z_{i} \right)+\varepsilon_{ij}$ | (7) |
| --- | --- |

In this model, the cost of plasticity within a given environment is detected as a negative value of the regression coefficient (β*_2_*) of genotype relative environment-specific fitness on genotype plasticity (Scheiner & Berrigan, 1998; Dorn *et al.,* 2000; van Buskirk & Steiner, 2009). When β*_2_* was significant, we estimated additional production costs by using an extension of this model as:

| $w_{ij}=\alpha+{\beta_{1}*z}_{ij}+\beta_{2}*slope\left( z_{i} \right)+\beta_{3}{*z}_{e}*slope\left( z_{i} \right)+\varepsilon_{ij}$ | (8) |
| --- | --- |

The additional cost of plasticity is detected as a negative value of the regression coefficient of the interaction term (β*_3_*).

**Methods S3. Quantification of the interaction with pollinators and herbivores**

We identified the pollinators visiting the flowers of the Negratín population both during spring and summer. For this, we conducted flower visitor counts in each season. All surveys were carried out between 11:00 am and 5:00 pm. During these surveys, we recorded the insects visiting the flowers of each individual plant during 5 minutes. The same researcher did all surveys. We only recorded those insects contacting anthers or stigma and doing legitimate visits, at least during part of their foraging at flowers. Those insects who ate petals or thieving nectar without doing any legitimate visit were not recorded as pollinator but as herbivore (see below). In total we did 15 surveys each season regularly distributed from the beginning to the end of each season.

We identified the herbivores consuming the plants of the Negratín population both during spring and summer. To do this, we conducted counts of herbivores during each season, recording the insects eating on reproductive and vegetative tissue of each individual plant as well as the damage caused by herbivores. The surveys lasted 250 minutes and were done by the same researcher. These surveys were done the same days that we did pollinator surveys.

**Methods S4. Genetic analyses**

We sampled leaf tissues from all the one hundred individuals from the Negratín population. These samples were subsequently desiccated and preserved in silica gel until DNA extraction.

DNA was extracted using the GenElute Plant Genomic DNA Miniprep Kit (Sigma-Aldrich, St. Louis, MO, USA) following the manufacturer’s instructions. We amplified seven microsatellite loci (Mmo031, Mmo170, Mmo185, Mmo212, Mmo235, Mmo319, and Mmo393) with fluorescent-labelled primers using the protocols and primer sequences of Cuenot *et al.*, (2018). Amplicons were pooled in mixes with different fluorochromes and analyzed by capillary electrophoresis with an Applied Biosystems™ 3130 DNA Analyzer by the CIC-UGR (Centre for Scientific Instrumentation, Granada University). Genotypes were called using the microsatellite plugin of Geneious Prime ([www.geneious.com](http://www.geneious.com), Dotmatics, Inc). G-exact test, cluster and PCA analyses were performed in R using packages adegenet (Jombart, 2008), genepop (Rousset, 2008), and pegas (Paradis, 2010).

**Methods S5. Structural Equation Modelling determining the factors mediating summer flowering**

We used Structural Equation Modelling (SEM) with latent constructs (Grace, 2006) to explore the occurrence of complex relationships between the spring life history, resource-acquisitive strategy and stress caused by the action of antagonistic organisms and the probability of flowering during summer. We built an *a priori* over-identified saturated model with three latent variables: (i) *Leaf economics spectrum* of the plants during springtime identified by six manifest variables measured during spring in the plants (SLA, LDMC, ratio between C and N concentration in leaves, and concentration of P, K and Mg in leaves); (ii) *Herbivory* undergone by plants during spring by eighteen herbivore guilds (see Table S2); (iii) *Life history* of the plants describing the performance of the plants during spring, described by five variables (plant size, height, number of flowers, number of ovules and number of seeds). In this model, we connected leaf economics spectrum to herbivory and life history, herbivory to life history and life history to the probability of summer flowering.

All models were solved by minimising yield-parameter estimates through an iterative process that uses Generalized Least Squares shifting to Maximum Likelihood as discrepancy functions. We used Maximum-Likelihood Estimation (MLE) on the variance-covariance matrix to test the goodness of fit of the models and retained those models obtaining an appropriate goodness of fit (*P* >0.05, Grace, 2006). We then checked their Standardized Root Mean Square Residual (SRMR), their Root Mean Square Error of Approximation (RMSEA), and their Comparative Fit Index (CIF). SRMR and RMSEA < 0.05 indicates a good fit to data, and between 0.05 and 0.1 indicates an acceptable fit. CFI > 0.97 means that the fit is better compared to the independence model (Cangur & Ercan, 2015). Structural Equation Modelling was performed using the R package lavaan (Rosseel, 2012).

**Supplementary references**

Adams, D. C., & Otarola-Castillo, E. (2013). Geomorph: an R package for the collection and analysis of geometric morphometric shape data. *Methods in Ecology and Evolution*, 4, 393-399.

Arnold, P. A., Kruuk, L. E., & Nicotra, A. B. (2019). How to analyse plant phenotypic plasticity in response to a changing climate. *New Phytologist,* 222, 1235-1241.

Arnold, S. J., & Wade, M. J. (1984). On the measurement of natural and sexual selection: theory. *Evolution*, 38, 709-719.

Briscoe, A. D., & Chittka, L. (2001). The evolution of color vision in insects. *Annual Review of Entomology*, 46, 471-510.

Cangur, S., & Ercan, I. (2015). Comparison of model fit indices used in structural equation modeling under multivariate normality. *Journal of Modern Applied Statistical Methods,* 14, 14.

Chittka, L., Shmida, A., Troje, N., & Menzel, R. (1994). Ultraviolet as a component of flower reflections, and the colour perception of Hymenoptera. *Vision Research*, 34, 1489-1508.

Cuenot, Y., Gómez, J. M., González‐Megías. A., Pannell. J. R., & Torices, R. (2018). Characterization of microsatellite markers for *Moricandia moricandioides* (Brassicaceae) and related species. *Applications in Plant Sciences*. 6, e01172.

Dorn, L. A., Pyle, E. H., & Schmitt, J. (2000). Plasticity to light cues and resources in *Arabidopsis thaliana*: testing for adaptive value and costs. *Evolution*, 54, 1982-1994.

Dryden, I. L. (2018). *Package ’Shapes’*. R foundation for statistical computing. Vienna, Austria. Contributed package. Version 1.2.4. URL <http://www.R-project.org>.

Dryden, I. L., & Mardia, K. V. (2016). *Statistical shape analysis, with applications in R*. Second Edition. John Wiley and Sons, Chichester.

Endler, J. A., & Mielke, P. W. (2005). Comparing entire colour patterns as birds see them. *Biological Journal of the Linnean Society*, 86, 405-431.

Giurfa, M., Vorobyev, M., Brandt, R., Posner, B., Menzel, R. (1997). Discrimination of coloured stimuli by honeybees: alternative use of achromatic and chromatic signals. *Journal of Comparative Physiology* *A*, 180, 235-243.

Gómez, J. M., Perfectti, F., Armas, C., Narbona, E., González-Megías, A., Navarro, L., DeSoto, L., & Torices, R. (2020). Within-individual phenotypic plasticity in flowers fosters pollination niche shift. *Nature Communications*, 11, 4019.

Grace, J. B. (2006). *Structural equation modeling and natural systems*. Cambridge University Press.

Lande, R., & Arnold, J. A. (1983). The measurement of selection on correlated characters. *Evolution*, 37, 1210-1226.

Lynch, M., & Walsh, B. (1998). *Genetics and analysis of quantitative traits* (Vol. 1). Sunderland, MA, Sinauer Associates, 535-557.

Jombart, T. (2008). adegenet: a R package for the multivariate analysis of genetic markers. *Bioinformatics,* 24, 1403-1405.

Paradis, E. (2010). Pegas: an R package for population genetics with an integrated–modular approach. *Bioinformatics*, 26, 419-420.

Pérez-Harguindeguy, N., Díaz, S., Garnier, E., Lavorel, S., Poorter, H., Jaureguiberry, P., Bret-Harte, M. S., Cornwell, W. K., Craine, J. M., Gurvich, D.E. & *et al*. (2016). New handbook for standardised measurement of plant functional traits worldwide. *Australian Journal of Botany*, 61, 167-234.

Rice, S. (2004). *Evolutionary theory: mathematical and conceptual foundations*. Sinauer Associates.

Rosseel, Y. (2012). Lavaan: An R package for structural equation modeling. *Journal of Statistical Software*, 48, 1-36.

Rousset, F. (2008). Genepop’007: a complete re‐implementation of the genepop software for Windows and Linux. *Molecular Ecology Resources*, 8, 103-106.

Savriama, Y. (2018). A step-by-step guide for geometric morphometrics of floral symmetry. *Frontiers in Plant Science*, 9, 1433.

Savriama, Y., Gómez, J. M., Perfectti, F., & Klingenberg, C. P. (2012). Geometric morphometrics of corolla shape: dissecting components of symmetric and asymmetric variation in Erysimum mediohispanicum (Brassicaceae). *New Phytologist*, 196, 945-954.

Scheiner, S. M., Mitchell, R. J., & Callahan, H. S. (2000). Using path analysis to measure natural selection. *Journal of Evolutionary Biology,* 3, 423-433.

Schlager, S. (2017). Morpho and Rvcg – Shape Analysis in R. In G. Zheng, S. Li, G. Szekely, (Eds.), *Statistical shape and deformation analysis* (pp. 217-256). Academic Press, London.

Spaethe, J., Tautz, J., & Chittka L. 2001. Visual constraints in foraging bumblebees: Flower size and color affect search time and flight behavior. *Proceedings of the National Academy of Sciences USA*., 98, 3898–3903.7.

Stinchcombe, J. R., Dorn, L. A., & Schmitt, J. (2004). Flowering time plasticity in *Arabidopsis thaliana*: a reanalysis of Westerman and Lawrence. *Journal of Evolutionary Biology*, 17, 197-207.

Van Buskirk, J., & Steiner, U. K. (2009). The fitness costs of developmental canalization and plasticity. *Journal of Evolutionary Biology*, 22, 852-860.

Van Kleunen, M., & Fischer, M. (2005). Constraints on the evolution of adaptive phenotypic plasticity in plants. *New Phytologist*, 166, 49-60.

Van der Kooi, C. J., & Spaethe, J. (2022). Caution with colour calculations: spectral purity is a poor descriptor of flower colour visibility. *Annals of Botany*, 13, 1-9.8.

Wright, I. J., Reich, P. B., Westoby, M., Ackerly, D. D., Baruch, Z., Bongers, F., Cavender-Bares, J., Chapin, T., Cornelissen, J. H., Diemer, M. et al. (2004). The worldwide leaf economics spectrum. *Nature*, 428, 821-827.

Zelditch, M. L., Swiderski, D. L., & Sheets, H. D. (2012). *Geometric morphometrics for biologists: a Primer*. Academic Press.
